# Development of novel *Meju* starter culture using plant extracts with reduced *Bacillus cereus* counts and enhanced functional properties

**DOI:** 10.1038/s41598-017-09551-0

**Published:** 2017-09-12

**Authors:** Shruti Shukla, Juyeon Park, Jung Hyun Park, Jong Suk Lee, Myunghee Kim

**Affiliations:** 1 Department of Energy and Materials Engineering, Dongguk University-Seoul, 30 Pildong-ro 1-gil, Seoul, 04620 Republic of Korea; 2 Department of Food Science and Technology, Yeungnam University, Gyeongsan-si, Gyeongsangbuk-do 38541 Republic of Korea; 3 Department of Food, Nutrition and Cooking, Taegu Science University, Daegu, 41453 Republic of Korea

## Abstract

We developed a novel type of *Meju* starter culture using single and combined extracts of *Allium sativum* (garlic clove), *Nelumbo nucifera* (lotus leaves), and *Ginkgo biloba* (ginkgo leaves) to improve the quality and functionality of *Meju*-based fermented products. *Meju* samples fermented with plant extracts (10 mg/ml) showed phenolic contents of 11.4–31.6 mg/g (gallic acid equivalents). Samples of extracts (garlic clove, lotus leaves, ginkgo leaves and their combination) fermented with *Meju* strongly inhibited tyrosinase, α-glucosidase, and elastase activities by 36.43–64.34%, 45.08–48.02%, and 4.52–10.90%, respectively. Specifically, ginkgo leaves extract added to fermented *Meju* samples at different concentrations (1% and 10%) strongly inhibited tyrosinase, α-glucosidase, and elastase activities and exhibited a potent antibacterial effect against *Bacillus cereus* with a significant reduction in bacterial counts compared with the effects observed for garlic clove and lotus leaf added to *Meju* samples. Scanning electron microscopy revealed severe morphological alterations of the *B. cereus* cell wall in response to ginkgo leaf extracts. Gas chromatographic mass spectroscopic analysis of plant extract-supplemented *Meju* samples and control *Meju* samples identified 113 bioactive compounds representing 98.44–99.98% total extract. The proposed approach may be useful for the development of various fermented functional foods at traditional and commercial levels.

## Introduction

Korean soy foods are becoming increasingly widespread in the global market. *Kochujang* (fermented red pepper paste with soybean, flour, and glutinous rice) and fermented soybean paste products (*Doenjang* and *Chungkukjang*) were registered in the Codex Alimentarius in July 2009 and are now internationally accepted foods^1^. *Meju* is a Korean traditional starter culture used to ferment various Korean traditional sauces, such as *Doenjang*. *Meju* is prepared from cooked soybean blocks as the main ingredient. During the natural drying period, live microorganisms, such as bacteria and fungi (e.g., yeast) with enzymatic activities, are cultivated^2, 3^. Although *Meju* manufactured by traditional methods has a favorable taste and adds functionality to fermented soybean foods, its safety remains controversial because of the presence of naturally occurring microorganisms associated with traditional *Meju* fermentation. Therefore, several attempts have been made to develop *Meju* manufacturing methods ensuring improved food safety by utilizing effective and beneficial microorganisms as the starter culture^4^.

Many synthetic antioxidants such as butylated hydroxyl anisole and butylated hydroxyl toluene are effective and are used for industrial processing. However, these synthetic chemicals possess toxic properties that pose potential risks to human health and should be replaced with natural antioxidants^5^. Additionally, exposing the skin to ultraviolet (UV) light results in the activation of reactive oxygen species (ROS) such as singlet oxygen and the superoxide anion radical, which can attack tissues in the dermis or epidermis, thus causing skin pigmentation^5^. Elastin is an extracellular-matrix protein providing elasticity to connective tissues^6^. Elastase is a proteinase enzyme that catalyzes the degradation of elastin^7^. Therefore, inhibition of elastase activity may be used as a method for protecting against skin aging^8^. Because accumulation of excessive epidermal pigmentation leads to various dermatological disorders, such as melasma associated with aging, freckling, age spots, and sites of actinic damage, tyrosinase inhibitors have become increasingly popular as medications and cosmetics for preventing hyperpigmentation through inhibition of enzymatic oxidation^9^. Hence, compounds derived from natural sources capable of protecting against ROS-mediated damage may have potential applicability in the prevention and/or treatment of skin diseases. Thus, there is a need to identify compounds that inhibit tyrosinase activity. Phenolic and flavonoid compounds derived from herb spices have been reported to be associated with biological activities such as tyrosinase inhibitory and anti-cholinesterase effects^10^. α-Glucosidase inhibitors reduce the rate of carbohydrate digestion and delay carbohydrate absorption in the digestive tract. Therefore, α-glucosidase inhibitors have the potential to prevent the development of type 2 diabetes by lowering after-meal glucose levels^11^. Plants and microorganisms are known as rich sources of α-glucosidase inhibitors^11^.

*Bacillus cereus* (B. cereus) is commonly found in contaminated foods containing fermented soybeans, such as *Doenjang*. The South Korean food authority has reported that ingestion of more than 10^4^ colony-forming units (CFUs) of *B. cereus* per gram of fermented soybean products may cause food poisoning^12^. It is well known that Korean traditional soybean paste products such as *Meju* and *Doenjang* are made by natural fermentation driven by various fungi and bacteria. *Meju* produced in this manner may be a good growth medium for *B. cereus*, which is known to cause food poisoning and can affect the production of soybean paste^13^. Furthermore, it has been reported that *Meju* fermentation is closely associated with rice straw because rice straw remains directly in contact with soybeans in *Meju* during the fermentation process (used for hanging soybean bricks in a natural environment), which provides *Meju* with various types of natural microflora^14^. In a recent survey, Park *et al*.^15^ confirmed the toxin profile of *B. cereus* and *Bacillus thuringiensis* (*B. thuringiensis*) in Korean fermented soybean food. Because spores of *B. cereus* are highly resistant to various stressors (heat, cold, radiation, desiccation, and disinfectants) and show excellent adhesion to food surfaces, *B. cereus* contamination is difficult to control in the fermented soybean food industry^16^. Various methods for inactivating *B. cereus* have been reported as control strategies. The use of plant-based bactericidal agents has become the most popular method for controlling *B. cereus* counts in food products. In recent years, biological methods in combination with medicinal plants that reduce microbial contamination levels in foods and feeds have been applied.

One important problem faced in the food sector is that *B. thuringiensis* is closely related to *B. cereus*; however, it can be distinguished from pathogenic strains of *B. cereus* through its ability to produce enterotoxin. The pathogenic spectrum of *B. cereus* ranges from strains that are used as human probiotics to lethal, highly toxic strains^17^. *B. cereus* is thus complex, harboring strains differing considerably in terms of their economic and sanitary importance. It is therefore important to determine the extent to which pathogenic strains can be distinguished from non-pathogenic strains. However, at the chromosomal level, there are currently no specific markers for unambiguously differentiating pathogenic from non-pathogenic strains. The pathogenicity of *B. cereus* depends on its ability to colonize and persist in the host and subsequently invade tissues. Kamar *et al*. (2013) reported that food poisoning and clinical strains can be differentiated from non-pathogenic strains based on host colonization phenotypes^17^. However, in the present work, we focused on determining how to reduce or eliminate *B. cereus* counts by considering a broad spectrum in which all identified strains might exhibit pathogenic characteristics.

*Ginkgo biloba* (G. biloba), *Allium sativum* (A. sativum), and *Nelumbo nucifera* (N. nucifera) are widely reported to have several biological activities, including antimicrobial, anti-proliferative, hepato-protective, and anti-angiogenic effects^18–20^. Based on these functional and biological activities, we aimed to develop a new strategy by using a combination of these plant extracts (*G. biloba*, *A. sativum*, and *N. nucifera*) at different concentrations (1% and 10% and/or a mixture of these extracts in a ratio of 1:1:1) for the production of soybean-based food products with improved quality. In our preliminary study, different concentrations of plant extracts (0.1%, 1%, 5%, and 10%) were tested for their antimicrobial efficacy against different food-borne pathogenic bacteria, including *B. cereus* strains that grow during *Meju* fermentation. Based on their potent anti-microbial efficacy during fermentation, two effective concentrations (1% and 10%) of plant extracts and a mixture of these extracts in a ratio of 1:1:1 were selected for the production of *Meju* samples.

Although production of commercial *Doenjang* products has been achieved using herbal products such as garlic and dried lotus leaves, in terms of health benefits, no systematic studies on *Doenjang* have been conducted on these plant extracts individually or in combination. Therefore, the aim of this study was to develop various types of novel *Meju* products by single and/or multiple inoculation of ethanolic extracts of various medicinal plants: *N. nucifera* (lotus) leaves, *G. biloba* (ginkgo) leaves, and *A. sativum* (garlic) cloves. Furthermore, we evaluated total phenolic content and α-glucosidase inhibitory effects as well as the anti-bacterial, anti-tyrosinase, and anti-elastase activities of the *Meju* samples created using single or multiple inoculation of ethanolic extracts derived from medicinal plants to confirm their biological and nutritional properties and quality characteristics. Furthermore, gas chromatography-mass spectrometry (GC-MS) analysis was performed on the *Meju* samples to evaluate their chemical composition.

## Materials and Methods

### Chemicals and reagents

Folin-Ciocalteu’s reagent, gallic acid, quercetin, mushroom tyrosinase, 3,4-dihydroxy-L-phenylalanine (DOPA), *p*-nitrophenyl-α-D-glucopyranoside, yeast α-glucosidase, elastase, aluminum chloride, sodium carbonate, hydrochloric acid, sodium chloride, sodium phosphate, methanol, ethanol, *p*-iodonitrotetrazolium violet, glucose, Tris base, potassium phosphate monobasic, potassium phosphate dibasic, kojic acid, ursolic acid, *N*-methyl-*N*-(trimethylsilyl)-trifluoroacetamide, methoxyamine hydrochloride, and pyridine were purchased from Sigma–Aldrich (St. Louis, MO, USA). Brain heart infusion (BHI), nutrient agar (NA), and nutrient broth (NB) were purchased from Difco (Franklin Lakes, NJ, USA), and mannitol-egg yolk-polymyxin agar was purchased from Oxoid Ltd. (Hampshire, UK). An analytical profile index (API kit) was acquired from (MYP) bioMerieux (Marcy l’Etoile, France), and 96-well microplates were obtained from SPL Life Sciences (Pocheon-si, Gyeonggi-do, Korea). Acarbose was purchased from Tokyo Chemical Industries (Toshima, Tokyo, Japan).

### Bacterial strain

*B. cereus* (KCCM 40935) used in this study was purchased from the Korean Culture Center of Microorganisms (KCCM; Seoul, Korea). Bacteria were cultured in NB for 18 h at 37 °C in a shaking incubator (150 rpm) and stored at −20 °C in glycerol stock form.

### Plant materials and preparation of extracts

All selected plant materials, including lotus leaves, ginkgo leaves, and garlic cloves were collected locally in Gyeongsan City, Korea, after which they were washed, cut, and stored at −20 °C for further processing. For preparation of ethanolic extracts, dried plant materials (100 g) were extracted with a 20-fold volume of 70% ethanol for 3 h at 65 °C. The extracts were filtered through Whatman No. 2 filter paper (Advantec; Tokyo, Japan). Filtrates of the ethanolic extracts were concentrated using a vacuum evaporator, freeze-dried, and stored at −20 °C for further use.

### Production of *Meju* fermented with plant extracts

*Meju* was produced by combining different concentrations of individual ethanolic extracts derived from different medicinal plants and their combinations. The plant extract ratios in *Meju* are summarized in Table 1. To produce *Meju*, soybeans (as per the required amount in a *Meju* product) were steamed in a suitable pot and crushed, after which the ethanolic extract of each plant species (lotus leaves, ginkgo leaves, and garlic cloves) and its mixture (1:1:1 ratio) were mixed at 1% (w/w) and 10% (w/w) concentrations in a square stainless-steel plate. All the mixtures were molded into a brick shape, hung with rice straw, and allowed to ferment naturally for 60 days. Traditional *Meju* fermented without ethanolic extracts of medicinal plants served as a control. All *Meju* samples were prepared in triplicate (Figure S1).

**Table 1:** List of various *Meju* samples prepared with plant extracts.

Plant extract ratio and composition used for *Meju*	Abbreviation of *Meju* samples
*Meju* with garlic cloves extract at 1% concentration (w/w)	GAM1
*Meju* with garlic cloves extract at 10% concentration (w/w)	GAM10
*Meju* with lotus leaf extract at 1% concentration (w/w)	LOM1
*Meju* with lotus leaf extract at 10% concentration (w/w)	LOM10
*Meju* with ginkgo leaf extract at 1% concentration (w/w)	GIM1
*Meju* with ginkgo leaf extract at 10% concentration (w/w)	GIM10
*Meju* with mixture extract (1:1:1) at 1% concentration (w/w)	MIM1
*Meju* with mixture extract (1:1:1) at 10% concentration (w/w)	MIM10
*Meju* without extracts (traditional *Meju*)	CON

### Physicochemical properties of plant extract-supplemented *Meju* samples

The physicochemical (moisture, ash, and salt contents) or organoleptic properties (sensory effects) of the plant extract-supplemented *Meju* samples were determined by the methods of the Association of Official Analytical Chemists^21^ (AOAC, 2006). For pH measurement of each plant extract-supplemented *Meju* product, the samples were diluted 10-fold with distilled water and then homogenized, followed by filtration through Whatman No. 2 filter paper (Advantec, Tokyo, Japan). The pH level was measured using a pH meter (Orion 35 star pH Benchtop, Thermo Electron Corporation; Beverly, MA, USA).

### Extraction procedure

*Meju* samples (10 g each) prepared using the ethanolic plant extracts were subjected to reflux extraction with 200 ml of distilled water for 3 h at 70 °C, followed by filtration through Whatman No. 2 filter paper. The residue was again extracted twice with an equal volume of water for 3 h. The filtrate was then freeze-dried. The yields of extracts of *Meju* samples were in the range of 22.1–25.25%.

### Determination of total phenolic content

The total phenolic content of each *Meju* sample prepared from the ethanolic plant extracts was determined using Folin-Ciocalteu’s reagent with gallic acid as a standard phenolic compound^22^. Briefly, 20 µl of each diluted *Meju* sample extract (1 mg/ml) was added to 100 µl of Folin-Ciocalteu’s reagent. After 3 min, 80 ml of a 10% aqueous sodium carbonate solution was added to the mixture. The solution was allowed to stand for 1 h at room temperature (RT), and absorbance of the resulting blue mixture was measured at 765 nm against a blank containing only the extraction solvent (200 µl). Total phenolic content was calculated as gallic acid equivalents (GAE) via the calibration curve constructed using the gallic acid standard solution and expressed in mg GAE/g dry mass.

### Determination of total flavonoid content

The total flavonoid content of each *Meju* sample prepared from ethanolic plant extracts was determined by the colorimetric method^23^. Briefly, 100 μl of each extract or standard reagent and 400 μl of ethanol were mixed with 500 μl of a 2% AlCl_3_ solution (diluted in distilled water). After 1 h of incubation at RT, absorbance was measured at 430 nm. Quercetin served as a reference compound to generate a standard curve, and results were expressed in milligrams of quercetin equivalents (mg QE/g dry mass).

### Determination of tyrosinase inhibitory activity of *Meju* samples

Tyrosinase inhibitory activity of *Meju* samples supplemented with plant extracts was determined by a method described previously^24^ with some modifications. Briefly, different concentrations (5, 10, 20, or 50 mg/ml) of each *Meju* sample (70 µl each) were separately added to a reaction mixture containing 40 µl of a 10 mM DOPA solution, 50 µl of 0.175 M sodium phosphate buffer (pH 6.8), and 40 µl of a mushroom tyrosinase solution (110 U/ml). The reaction mixture was allowed to incubate at 37 °C for 2 min, after which the amount of dopachrome produced was measured at 475 nm on a microtiter plate reader. Kojic acid (62.5, 125, 250, 500 and 1000 µg/ml) was used as a standard compound for a positive control. The percentage of tyrosinase inhibition was calculated as follows:

1 $${\rm{Inhibition}}\,( \% )=[1-({{\rm{A}}}_{{\rm{sample}}}{/{\rm{A}}}_{{\rm{blank}}})\times 100]$$

### Determination of α-glucosidase inhibitory activity of *Meju* samples

α-Glucosidase inhibitory activity of *Meju* samples supplemented with plant extracts was evaluated according to a chromogenic method^25^, with minor modifications. Briefly, various concentrations (5, 10, 20, and 50 mg/ml) of *Meju* samples (50 µl) and 100 µl of α-glucosidase (1.0 U/ml) dissolved in 0.1 M phosphate buffer (pH 6.9) were mixed in a 96-well microplate and incubated at 25 °C for 10 min. After pre-incubation, 50 µl of *p*-nitrophenyl-α-D-glucopyranoside (5 mM) in 0.1 M phosphate buffer (pH 6.9) was added into each well as a substrate solution. The reaction mixture was incubated at 25 °C for 5 min. Absorbance was recorded using a microtiter plate reader at 405 nm before and after incubation with a *p*-nitrophenyl-α-D-glucopyranoside solution and compared with that of a control containing only 50 µl of the buffer. Acarbose at various concentrations (31.3, 62.5, 125, 250, 500, 1000, and 2000 µg/ml) was used as a standard compound. Each experiment was conducted in triplicate, and the enzyme inhibitory rates of the samples were calculated as follows:

2 $${\rm{Inhibition}}\,( \% )=[1-({{\rm{A}}}_{{\rm{sample}}}{/{\rm{A}}}_{{\rm{blank}}})\times 100]$$

### Determination of elastase inhibitory activity

Elastase inhibitory activity of *Meju* samples supplemented with plant extracts was determined according to a previously reported method^26^, with minor modifications. For this purpose, 200 μl of 0.05 M Tris-HCl buffer (pH 8.6) and 20 μl of a *Meju* sample analyzed at various concentrations (5, 10, 20, and 50 mg/ml) were mixed and incubated for 15 min. Then, 10 μl of 2.5 U/ml elastase (optimum reactivity of the enzyme) was added, after which the reaction mixture was incubated for another 15 min; the absorbance was then measured at 410 nm. Ursolic acid at various concentrations (100, 250, 500, and 1000 and 10000 µg/ml) served as a standard compound. The elastase inhibitory rate was calculated as follows:

3 $${\rm{Inhibition}}\,( \% )=[1-({{\rm{A}}}_{{\rm{sample}}}{/{\rm{A}}}_{{\rm{blank}}})\times 100]$$

### Determination of *B. cereus* counts in the *Meju* samples

To analyze *B. cereus* cell numbers, 10 g of each *Meju* sample supplemented with plant extract(s) was homogenized in a blender in 90 ml of a 0.85% sterile NaCl solution and serially diluted to 1:10^1^–1:10^6^ with 0.01 M potassium phosphate buffer (pH 7.2). Enumeration of *B. cereus* cells was performed by spreading 0.2 ml of each diluted sample onto the surface of mannitol-egg yolk-polymyxin agar plates (a total of five media plates), which were incubated at 30 °C for 24 h^27^. Pink colonies with transparent zones were counted, and further confirmatory tests were performed by streaking the observed colonies onto the NA medium; the grown colonies were confirmed using the API kit. To determine the enterotoxin-producing capability of each isolate, a loopful of each bacterial colony was inoculated into 10 ml of the BHI medium containing 0.1% glucose with incubation for 16–18 h at 36 °C before performing a *Bacillus* diarrhea enterotoxin microscopic examination assay (test for protein toxin crystals) to differentiate bacteria from the *B. thuringiensis* strain^28^. Confirmatory tests for counting *B. cereus* cells were carried out by molecular identification at Solgent Co. (Doejon, Korea).

### Inhibitory spectrum of the tested plant extracts against *B. cereus*

#### Growth and preparation of the bacterial strain

*B. cereus* was grown in NB at 37 °C for 18–24 h. After proper bacterial growth, the bacterial culture was diluted with peptone water to adjust it to the proper concentration (10^7^ CFU/ml) and was exposed to the extract solution (1% and 10%) of *A. sativum*, *G. biloba*, or *N. nucifera*. Prior to scanning electron microscopy (SEM) analysis, we also performed other inhibitory assays to confirm the inhibitory effects of the plant extracts. The details of the method are described in Supplementary Information Section 1.

#### SEM analysis

SEM was conducted to determine the effects of all tested plant extracts on the morphology of the *B. cereus* under study at the minimum inhibitory concentration (MIC). Briefly, the bacterial cells (10^7^ CFU/ml) of the control and treatment groups were washed with 0.05 M phosphate buffer (pH 7.0), followed by centrifugation (4000 × *g*, 10 min), and the cells were first fixed in 2.5% (w/v) glutaraldehyde at room temperature for 2 h. Secondary fixation was conducted in a 1% (v/v) arsenic acid solution at 4 °C overnight. Sample groups were dehydrated by increasing the concentration of ethanol and freeze-dried. Control samples were prepared without the addition of plant extract samples. To examine morphological changes, a published SEM protocol^29^ was modified. Finally, each bacterial sample was sputter-coated with gold in an ion coater for 2 min, followed by examination under a scanning electron microscope (Hitachi; Hitachi City, Japan).

### GC-MS profile of bioactive compounds in *Meju* samples

#### Extraction of bioactive compounds from Meju

For GC-MS analysis, different *Meju* samples were subjected to extraction as suggested by Namgung *et al*.^30^. Briefly, *Meju* samples (5 g each) were extracted using 50 ml of 80% methanol in water (v/v) at 70 °C for 30 min, followed by filtration through Whatman No. 2 filter paper. The residue was again extracted twice with the same volume of 80% methanol for 30 min, followed by filtration. The filtrate was then concentrated under reduced pressure and diluted up to a volume of 10 ml with 80% methanol, followed by centrifugation (10,000 rpm; 10 min). Next, the transparent upper layer of each extract (1 ml) was transferred into a glass vial, and methanol was evaporated under a constant stream of nitrogen at 40 °C, followed by derivatization.

#### Derivatization of Meju sample extracts

For derivatization, 50 µl of the methoxyamine hydrochloride reagent (50 mg dissolved in 1 ml of pyridine) was added to each methanol-extracted *Meju* sample in an extraction vial; the components were mixed and kept at 40 °C for 60 min. Then, 100 µl of the *N*-methyl-*N*-(trimethylsilyl)-trifluoroacetamide reagent was added into each vial, followed by incubation at 40 °C for 45 min^31^. Finally, the derivatized samples were filtered using syringe filters (0.45 µM) and kept in GC vials. Bioactive compounds in *Meju* samples were identified on a GC-MS system (Agilent; Santa Clara, CA, USA).

#### GC-MS conditions and analysis

Chromatography was carried out on a Hewlett-Packard (Palo Alto, CA, USA) fused silica column DB-5 MS UI (30 m length, 0.25 mm inner diameter, and 0.25 mm thickness). The GC-MS conditions were reported elsewhere^31^. The relative proportions of the extract constituents were expressed as percentages by peak area normalization. Extract components were identified based on GC retention time relative to computer matching of mass spectra using Wiley and National Institute of Standards and Technology libraries for the GC-MS system.

#### Statistical analysis

The data were expressed as the mean ± standard deviation of three independent experiments and subjected to one-way analysis of variance and Student’s *t* test. Data with *P* values < 0.05 were considered statistically significant. IC_50_ values (concentration required for 50% inhibition) were calculated using the formula Y = 100 × A1/(X + A1), where A1 = IC_50_, Y = response, X = inhibitory concentration (linear regression analysis).

### Data availability

The authors declare that all the other data supporting the finding of this study are available within the article and from the corresponding author on reasonable request.

## Results and Discussion

### Production of *Meju* fermented with plant extracts

All *Mej*u samples were prepared in two different sets to determine the variability of the results, and the microflora in each lot was analyzed. As presented in Figure S1, the presence of natural microflora (fungal growth) in *Meju* samples fermented for 60 days in a natural environment with and without plant extracts confirmed the fermentation of *Meju* samples (control as well as plant extract-supplemented samples).

### Physico chemical properties of *Meju* samples

To assess the acceptability of the plant extract-supplemented *Meju* products, physico chemical properties were analyzed by the standard AOAC methods^21^. Ash content indicates the amount of mineral salts present in the diet^32^. The *Meju* samples supplemented with plant extracts after 60 days of natural fermentation contained ash contents ranging from 11.85 ± 1.02% to 14.37 ± 1.82%. The control *Meju* sample without plant extracts, however, showed an ash content of 13.82 ± 1.63%. No significant difference in ash content was observed among all the plant extract-supplemented *Meju* samples. With respect to moisture content, the plant extract-supplemented *Meju* samples showed a moisture content ranging from 54.46 ± 1.65% to 64.48 ± 1.38%, whereas control samples showed a moisture content of 55.47 ± 0.16%. As the fermentation period increased, the moisture content decreased in all *Meju* samples. All the plant extract-supplemented *Meju* samples showed salt contents ranging from 7.51 ± 1.52% to 8.23 ± 3.11%, whereas in the control *Meju*, the salt content was 10.87 ± 1.44%. The results of pH analysis revealed that there was no significant difference in pH among different samples after different fermentation periods. The pH levels of all the *Meju* samples prepared with plant extracts were in the range 5.05–6.01. Traditionally, the pH level of fermented *Meju* samples (without plant extracts) is 6.3. It is a well-established phenomenon that fermented foods with pH levels below 4 are usually safe because most of the pathogens are unable to survive in this pH range^33^. Based on the parameters obtained, we may obtain official approval of our plant extract-supplemented *Meju* samples because they have physicochemical and organoleptic properties similar to those of originally produced traditional *Meju* samples without plant extracts.

### Total phenolic and flavonoid contents

There is great interest in natural phenolic and flavonoid antioxidants because of their presence in edible plants, their health benefits, and their possible use as natural food preservatives^34^. Raw materials such as soybeans and plants are rich in various bioactive phytochemicals (phenolics and flavonoids). Therefore, in the present study, we analyzed the total phenolic and flavonoid contents of *Meju* samples supplemented with plant extracts and compared the values with those of traditional *Meju* samples (Table 2). The total phenolic content of *Meju* samples containing 10% lotus leaf extract (LOM10) showed the highest value, 31.6 ± 0.51 mg GAE/g. On the other hand, the flavonoid contents of *Meju* samples supplemented with 10% plant extracts (GAM10, LOM10, GIM10, and MIM10) were 7.13 ± 0.07, 23.75 ± 0.62, 7.90 ± 0.14, and 11.69 ± 0.19 mg QE/g, respectively, and were higher than the flavonoid content of the control *Meju* (7.47 ± 0.28 mg QE/g) prepared without any plant extracts (Table 2). However, some researchers have reported that the flavonoid content of fermented soybean products may vary owing to differences in the raw materials used during fermentation^35, 36^. Lin *et al*.^37^ suggested that the starter culture or microflora can affect the antioxidant activity of soybeans after fermentation. Nam *et al*.^38^ reported that flavonoid content can be significantly affected by the soybean cultivar and fermentation period. These results suggest that higher antioxidant activity may be due to the presence of phenolic and flavonoid compounds in *Meju* samples fermented with various plant extracts. Hence, extracts with a high polyphenolic content likely have strong antioxidant activity.

**Table 2:** Total phenolic and flavonoid contents and inhibitory activities of tyrosinase, α-glucosidase, and elastase (IC_50_ values) of *Meju* samples with and without added plant extracts.

*Meju* samples	Total phenolic content (mg GAE/g)	Total flavonoid content (mg QE/g)	IC_50_ values (mg/ml)		
			Tyrosinase inhibitory activity	α-Glucosidase inhibitory activity	Elastase inhibitory activity
GAM1	11.4 ± 0.26	7.63 ± 0.12	296.74	8.95	199.93
GAM10	11.7 ± 0.94	7.13 ± 0.07	43.54	21.82	234.10
LOM1	14.15 ± 0.22	8.66 ± 0.32	150.01	5.83	199.70
LOM10	31.6 ± 0.51	23.75 ± 0.62	33.36	4.87	208.03
GIM1	14.0 ± 2.01	6.72 ± 0.06	84.75	21.82	240.10
GIM10	19.74 ± 0.58	7.90 ± 0.14	32.80	10.27	259.72
MIM1	14.1 ± 1.35	7.97 ± 0.39	178.98	11.96	346.97
MIM10	21.7 ± 0.62	11.69 ± 0.19	76.98	2.86	1364.86
CON	14.3 ± 1.58	7.47 ± 0.28	62.35	10.48	171.86
Standard			0.141	0.70	0.85

Results are presented as the mean ± standard deviation of three experiments.

### Tyrosinase inhibitory activity

**Figure 1:**
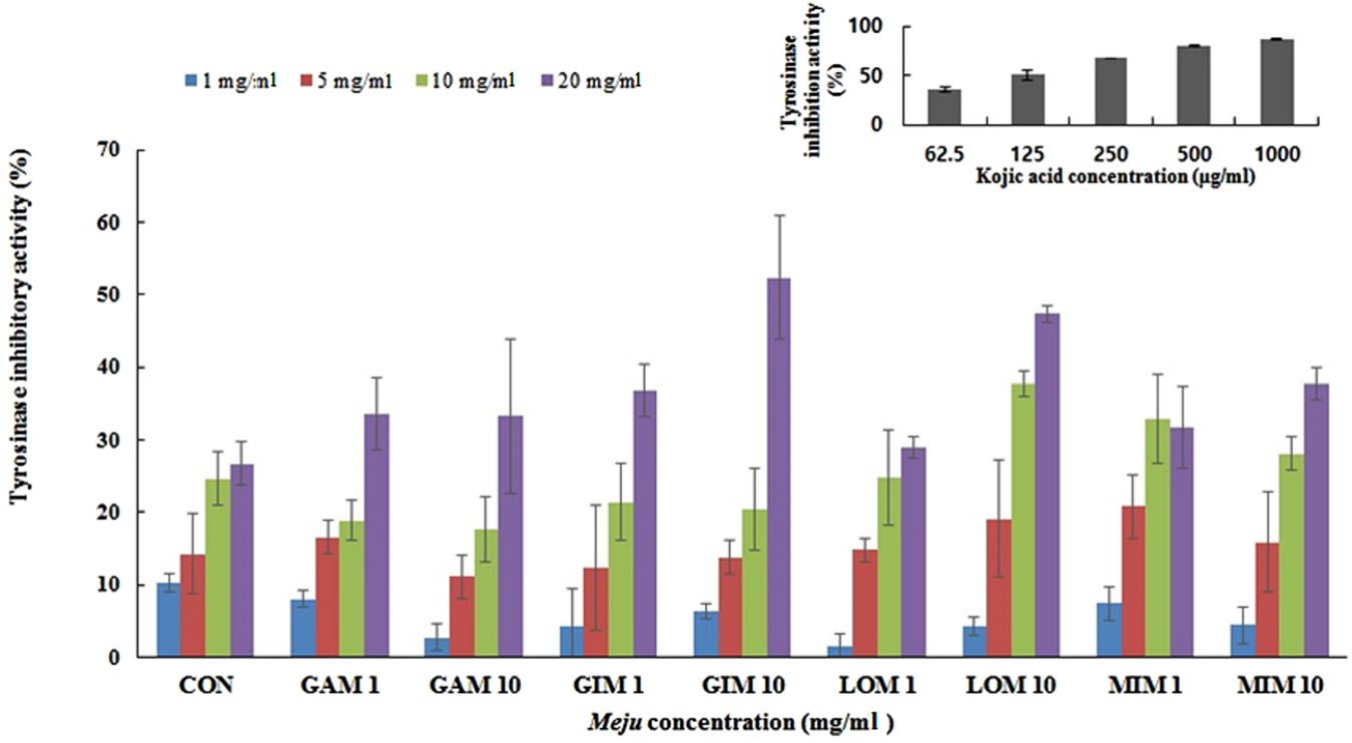
Tyrosinase inhibitory activities of *Meju* samples supplemented with plant extracts. Values are the means of six measurements. Error bars represent standard deviations. The coefficient of variation for tyrosinase inhibitory activities (n = 6) is <15%.

Tyrosinase is a major enzyme of the melanin synthesis pathway in melanocytes. Because melanin causes dark spots and freckles on the skin^39, 40^, inhibition of tyrosinase could be an important strategy for blocking melanogenesis^40^. Commercial tyrosinase inhibitors such as kojic acid, arbutin, ascorbic acid derivatives, retinoic acid, and azelaic acid are used as ingredients in cosmetics to prevent hyperpigmentation owing to their skin-whitening efficacy^39^. In the present study, *Meju* samples supplemented with various plant extracts individually or in combination at a concentration of 20 mg/ml showed tyrosinase inhibitory effects ranging from 36.84 ± 3.61% to 52.34 ± 3.99%, whereas the inhibitory effect of the control *Meju* sample (with no added plant extracts) (20 mg/ml) was 41.25 ± 4.53% (Fig. 1). Kojic acid at various concentrations (62.5, 125, 250, 500 and 1000 µg/ml) showed tyrosinase inhibitory activities of 36.35 ± 0.73%, 45.67 ± 0.64%, 67.28 ± 0.19%, 79.41 ± 3.80%, and 86.34 ± 1.94%, respectively. The concentrations of tested *Meju* samples supplemented with plant extracts individually or in combination and of the control *Meju* sample required for 50% inhibition were 32.80–296.74 mg/ml and 62.35 mg/ml, respectively, whereas a concentration of 0.141 mg/ml was required for kojic acid (Table 2).

The majority of natural tyrosinase inhibitors derived from functional food products (food/formulations) consist of phenolic or flavonoid components^39^. In addition, the tyrosinase inhibitory activity may depend on the hydroxyl groups of phenolic compounds, which form hydrogen bonds with the enzyme’s active site, thereby causing steric hindrance, conformational changes, and ultimately suppression of enzymatic activity^41^. Thus, the phenolics present in our *Meju* samples may play a major role in inhibiting tyrosinase activity. Polyphenols may also be used as depigmentation agents because of their structural similarity to tyrosine, a substrate of tyrosinase^41^. In addition, antioxidants inhibit pigmentation by various mechanisms, including scavenging of ROS and reactive nitrogen species as well as reduction of *o*-quinones or other intermediates of melanin biosynthesis, thus delaying oxidative polymerization^42^. Therefore, polyphenolic compounds are partially responsible for the efficacy of substances used as whitening agents in skin care products. Similarly, dose-dependent tyrosinase inhibition is observed in fermented soybean broth owing to increased concentrations of total phenolics^43^. Chai *et al*.^44^ examined the tyrosinase inhibitory activities of various fermented-soybean-based food products and reported that almost all samples at a particular concentration exerted similar inhibitory activities, except for a *Meju* sample showing very high activity: from 5 mg/ml to 20 mg/ml. Our results suggest that *Meju* samples supplemented with plant extracts are rich in phenolic and flavonoid compounds and may be applicable to the treatment of melanin-related pigmentation as skin-whitening agents.

### Elastase inhibitory activity

Elastin is a major component of skin connective tissue and plays an important role in maintaining skin elasticity^39^. Elastin also participates in the formation of a network with collagenous fibers under the epidermis^45^. Elastase hydrolyzes peripheral and structural proteins in dermal connective tissue and has a strong ability to degrade elastin^45^. Because decomposition of elastin results from the activation of elastase caused by UV irradiation or ROS formation, inhibition of elastase activity could be a therapeutic target for protection against elastin-induced skin aging^46^. The active ingredient responsible for this inhibitory activity is believed to be phenolics and flavonoids present in plant extracts^47^.

**Figure 2:**
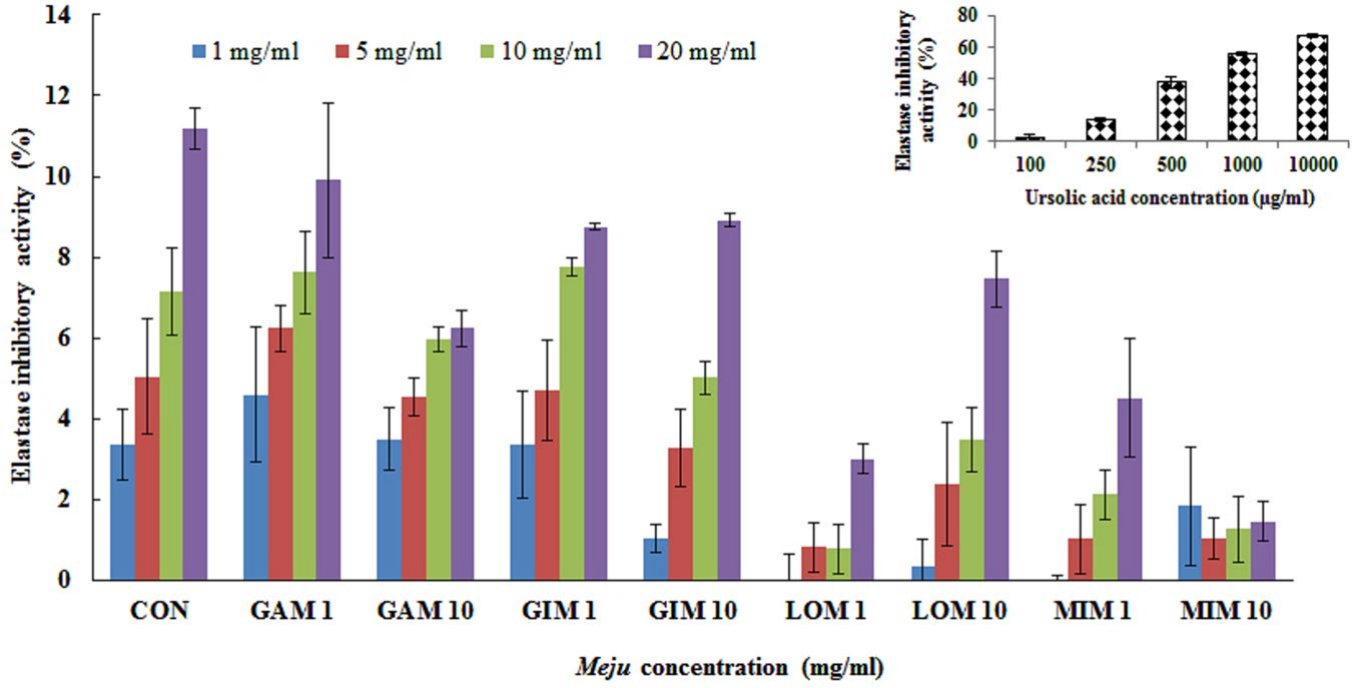
Elastase inhibitory activities of *Meju* samples supplemented with plant extracts. Values are the means of six measurements. Error bars represent standard deviations. The coefficient of variation for elastase inhibitory activities (n = 6) is <15%.

The elastase inhibitory activities of *Meju* samples supplemented with plant extracts individually or in combination and the activity of a *Meju* sample without plant extracts are shown in Fig. 2. In this assay, *Meju* samples supplemented with extracts individually and in combination yielded optimal results at 20 mg/ml. Notably, all the tested *Meju* samples (20 mg/ml) supplemented with plant extracts of garlic cloves, lotus leaves, and ginkgo leaves individually or in combination (1:1:1) exerted elastase inhibitory activities of 9.90 ± 1.92%, 8.74 ± 1.07%, 3.01 ± 1.35%, and 5.52 ± 1.01%, respectively, although their values were lower than the activity of the control *Meju* sample (11.18 ± 1.51%; Fig. 2). In this assay, the IC_50_ values of all the experimental and control *Meju* samples were 199.70–1364.86 mg/ml and 171.86 mg/ml, respectively. On the other hand, the IC_50_ value of the positive control (ursolic acid) for elastase inhibition was 0.85 mg/ml (Table 2). Although the *Meju* samples supplemented with plant extracts did not show significant elastase inhibitory activities for possible skin improvement, they showed positive results regarding melanin-reducing (tyrosinase inhibitory) activity.

*In vitro* studies on both purified elastases and cultured fibroblasts showed that soybean extracts can affect the extracellular matrix and inhibit enzymatic activities of several elastases^48^. Based on these studies, it can be inferred that all our *Meju* samples fermented with plant extracts as well as the control *Meju* sample may have beneficial effects on human skin and certain dermatological disorders because of the enhanced production of bioactive compounds (phenolics and flavonoids) during fermentation. The increasing use of phenolic and flavonoid compounds in the cosmetic industry could be attributed to the compounds’ health benefits, including antioxidant properties and skin-improving effects^49^. These findings suggest that the consumption of *Meju* supplemented with plant extracts may have positive effects and be useful for the treatment of skin pigmentation.

### α-Glucosidase inhibitory activity

**Figure 3:**
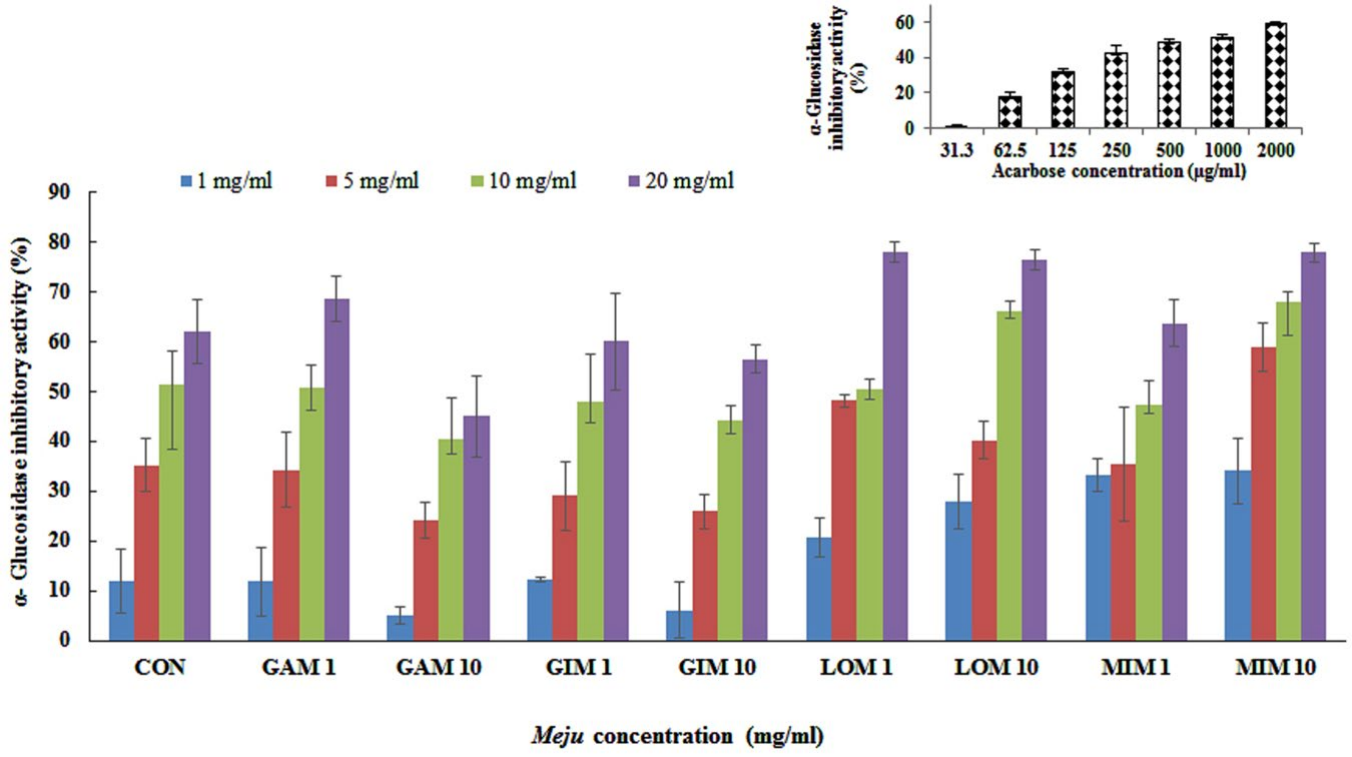
α-Glucosidase inhibitory activities of *Meju* samples supplemented with plant extracts. Values are the means of six measurements. Error bars represent standard deviations. The coefficient of variation for α-glucosidase inhibitory activities (n = 6) is <15%.

The application of *Meju* samples fermented with herbal extracts for treating diabetes remains largely unexplored; this situation has prompted some scientists to develop novel agents with inhibitory effects on intestinal glucosidases. The *in vitro* α-glucosidase inhibitory activities of *Meju* samples are summarized in Table 2 and Fig. 3. The α-glucosidase inhibitory activities of all the tested *Meju* samples fermented with extracts of garlic cloves, lotus leaves, and ginkgo leaves individually or in combination (1:1:1) were determined using *p*-nitrophenyl-*α*-D-glucopyranoside as a substrate and compared with the activity of the control *Meju* sample (without addition of plant extracts; Table 2). Acarbose served as a standard compound. In this assay, the percentages of α-glucosidase inhibition shown by *Meju* samples fermented with individual plant extracts were similar to that of *Meju* samples fermented with a combination of extracts (1:1:1), indicating a synergistic or additive interaction between the plant extracts and *Meju* samples. The control *Meju* sample showed a lower α-glucosidase inhibitory activity than did the other *Meju* samples. The IC_50_ values of all the tested *Meju* samples fermented with ethanolic extracts derived from garlic cloves, lotus leaves, and ginkgo leaves individually or in combination (1:1:1) ranged from 2.86 mg/ml to 21.82 mg/ml (Table 2). The control *Meju* sample showed a higher IC_50_ value (10.48 mg/ml) than did the *Meju* sample supplemented with lotus leaf extract (LOM1 and LOM10: 5.83 mg/ml and 4.87 mg/ml, respectively; Table 2). On the other hand, the IC_50_ value of the standard compound (acarbose) for α-glucosidase inhibition was 0.70 mg/ml (Table 2). In addition, all the tested *Meju* samples (20 mg/ml) supplemented with a 1% plant extract of garlic cloves, lotus leaves, and ginkgo leaves individually or in combination exerted considerable α-glucosidase inhibitory activities: 68.66% ± 4.47%, 72.12% ± 9.71%, 77.94 ± 2.04%, and 63.76 ± 4.60%, respectively. In contrast, this inhibitory activity was slightly lower for the control *Meju* sample (60.07 ± 6.53%; Fig. 3). The inhibitory activities of *Meju* samples fermented with plant extracts against α-glucosidase may be due to the increased glycoside content of soybeans caused by either fermentation or the addition of plant extracts. Glycosides consist of sugars that may be structurally similar to the carbohydrate substrate of α-glucosidase^50^. *Meju* samples fermented with plant extracts were found to have lower IC_50_ values than the control *Meju* sample because their active chemical compounds did not undergo fractionation and may synergistically inhibit α-glucosidase^51^. Despite several studies on *Doenjang* samples^31, 35, 43, 44, 51^ with *Meju* as a fermentation starter, there has been no investigation of enzyme-based α-glucosidase inhibition in normal *Meju* samples. In addition, there are various scientific reports on the health benefits of garlic cloves, lotus leaves, and ginkgo leaves, including anti-obesity and anti-diabetic effects^52, 53^, suggesting that *Meju* samples fermented with these plant extracts could be used to develop functional foods for the prevention of diabetes with enhanced biological activities.

### Reduction in *B. cereus* counts in *Meju* samples fermented with plant extracts

In this assay, all the selected plant extracts derived from garlic cloves, lotus leaves, and ginkgo leaves individually or in combination (1:1:1) were used to prepare *Meju* during fermentation to examine their ability to reduce *B. cereus* counts in samples of the soybean-based *Meju* starter culture. Results showed that *B. cereus* counts in *Meju* samples fermented with individual plant extracts of garlic cloves, lotus leaves, and ginkgo leaves or their combination were drastically reduced compared with those in the traditional *Meju* sample fermented without any plant extract. *Meju* samples fermented with individual plant extracts showed *B. cereus* counts ranging from 0 CFU/g to (1.0 ± 0.1) × 10^2^ CFU/g. On the other hand, the control *Meju* sample showed *B. cereus* counts ranging from (1.7 ± 0.01) × 10^3^ CFU/g to (1.8 ± 0.07) × 10^4^ CFU/g (Table 3). *Meju* samples fermented with a higher concentration (10%) of plant extracts inhibited the growth of *B. cereus* better than samples fermented with a low concentration (1%) of plant extracts (Table 3). To confirm that the bacterium found in the *Meju* samples (1% plant extract-supplemented *Meju* and control *Meju*) was *B. cereus*, colonies on MYP agar were identified using the API kit, and all the analyzed colonies tested positive for *B. cereus* with 99.9% ID and T-index values ranging from 0.37 to 0.62. These data confirmed how closely the profile corresponds to the taxon in question relative to all other taxa in the database, and how closely the profile corresponds to the most typical set of reactions for each taxon, respectively (Table 3). Moreover, among all the *Meju* samples fermented with plant extracts, the *Meju* samples that were fermented with the 1% and 10% *ginkgo* leaf extract showed a remarkable reduction in *B. cereus* counts (Table 3). Confirmatory tests for counted *B. cereus* cells were also performed by molecular identification. Results showed that all the colonies suspected of containing *B. cereus* tested positive for *B. cereus* with 100% similarity (data not shown).

**Table 3:** *Bacillus cereus* counts from *Meju* samples added with and without plant extracts.

*Meju* samples	*Bacillus cereus* (CFU/g) presumptive test (MYP^1^)	Confirmatory test (API^2^)			
		Test result	Identified strain	%ID*	T-index**
GAM1	(1.0 ± 0.10) × 10^2^	+	*Bacillus cereus*	99.9	0.62
GAM10	(2 ± 0.77) × 10^1^	+	*Bacillus cereus*	99.9	0.37
LOM1	(5 ± 1.41) × 10^1^	+	*Bacillus cereus*	99.9	0.62
LOM10	No colonies observed	−	−	−	−
GIM1	(1 ± 0.01) × 10^1^	+	*Bacillus cereus*	99.9	0.42
GIM10	No colonies observed	−	−	−	−
MIM1	(2.1 ± 0.10) × 10^1^	+	*Bacillus cereus*	99.9	0.59
MIM10	No colonies observed	−	−	−	−
CON	(1.7 ± 0.01) × 10^3^ − (1.8 ± 0.07) × 10^4^	+	*Bacillus cereus*	99.9	0.51

^1^Mannitol-egg yolk-polymyxin agar plate; ^2^Analytical profile index. *Percent similarity; that is, how closely the profile corresponds to the taxon relative to all other taxon in the database. **T-index; that is, how closely the profile corresponds to the most typical set of reactions for each taxon.

According to the abovementioned results, the addition of 10% ethanolic extracts of selected plants individually or in combination may be a feasible approach to controlling the growth of food-borne *B. cereus* in starter culture *Meju* samples. Lim and Lee^13^ observed a growth reduction of *B. cereus* in *Meju* samples prepared from licorice extracts at different concentrations. Although various methods for inactivating *B. cereus* have been developed, including ethanol, sodium chloride, and microwave methods^13, 54^, the application of plant extracts of natural origin to *Meju* samples appears to be the most effective method for controlling the growth of hazardous pathogens such as *B. cereus*. In addition, Lim and Lee^13^ produced licorice extracts to enhance *Meju* samples with respect to sensory attributes and consumer acceptability. In the present work, fermented soybean paste samples were approved by performing sensory examinations and other consumer acceptability tests, which were not elaborated when licorice extracts were incorporated into *Meju* samples. Furthermore, the Korean population is well aware of the use of a few of these plant extracts (garlic cloves and lotus leaves) in different types of fermented soybean products. These findings suggest that selected plant extracts derived from garlic cloves, lotus leaves, and ginkgo leaves individually or in combination can be utilized in conjunction with other conventional food safety measures to inhibit the growth of *B. cereus* in *Meju* samples.

### Confirmation of inhibitory effects of plant extracts against *B. cereus*

**Figure 4:**
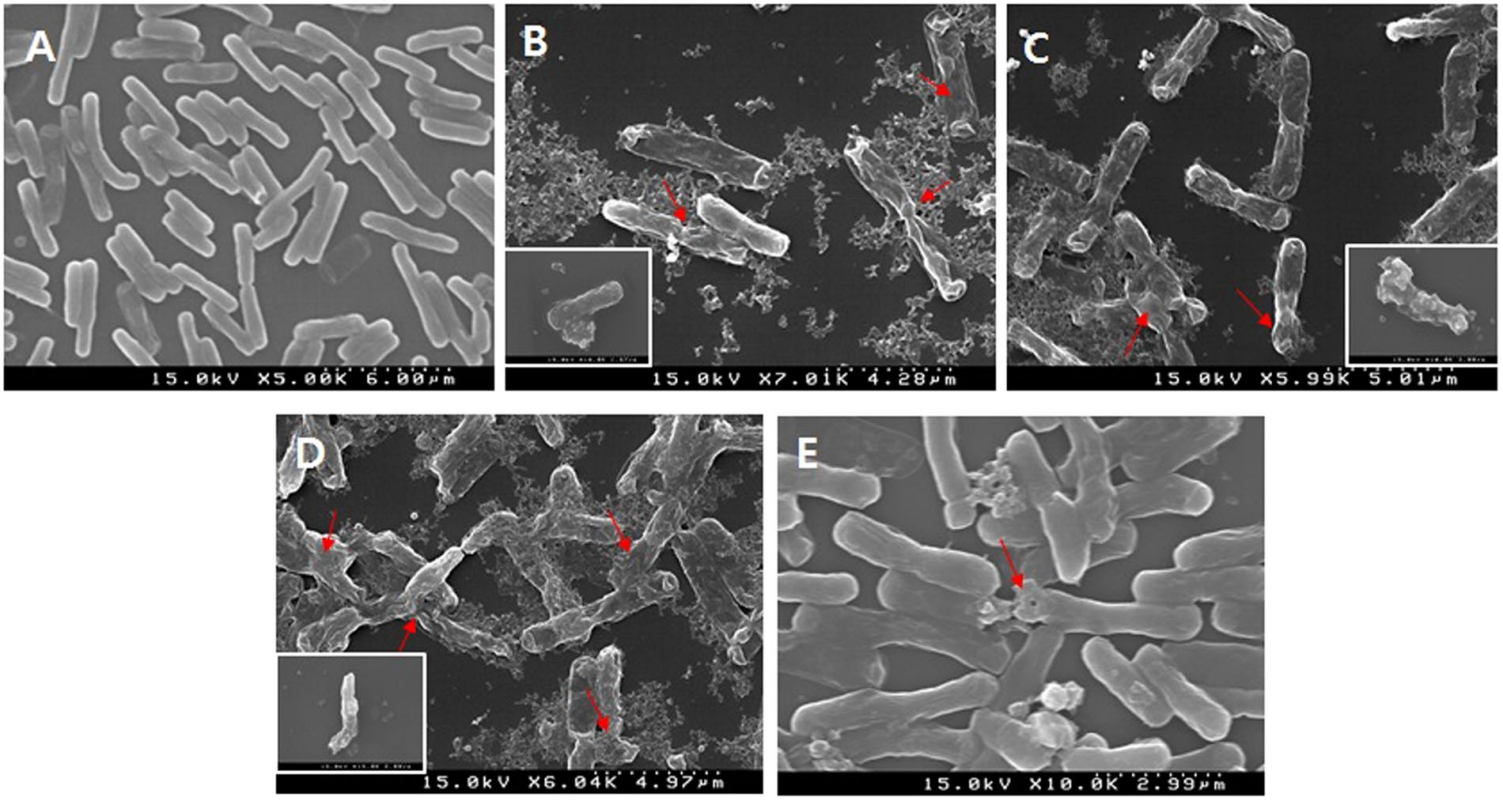
Scanning electron microscopy of *Bacillus cereus* treated with single or combined ethanolic extracts of lotus leaves, ginkgo leaves, and garlic cloves. (**A**) Without any treatment (showing regular and smooth surface of cells); *Bacillus cereus* cells treated with ethanolic extracts of lotus leaves (**B**), ginkgo leaves (**C**), garlic cloves (**D**), and combination of extracts in a ratio of 1:1:1 (all showing disruption, swelling, surface collapse, and cell lysis); (**E**) *A. sativum* (lesser cell lysis).

Physical and morphological alterations may induce deterioration of the cell wall surface of bacterial pathogens upon treatment with a suitable antimicrobial agent. Hence, SEM analysis was carried out to visualize the effects of these plant extracts individually and in combination on the morphology of *B. cereus* cells relative to a control group (Fig. 4). Ethanolic extracts of lotus leaves and ginkgo leaves revealed possible inhibitory effects, as confirmed by severe morphological alteration of the cell wall of *B. cereus*, leading to cell disruption and lysis (Fig. 4B and C). Furthermore, *B. cereus* cells treated with plant extracts showed severely damaged cell morphology, including disruption of the cell membrane, abnormal cell breaking, and swelling. In contrast, control cells of the pathogen tested (in the absence of any plant extract) showed regular and smooth surfaces (Fig. 4A). In our preliminary study, the *in vitro* inhibitory activities of ethanolic extracts of garlic cloves, lotus leaves, ginkgo leaves added individually and in combination (1:1:1) against *B. cereus* were qualitatively and quantitatively assessed by the presence of inhibition zones and MIC values (Supplementary Section 1). Results showed that extracts derived from ginkgo leaves, lotus leaves, and combinations thereof had possible inhibitory effects on *B. cereus* (data not shown). Several researchers have also demonstrated the anti-bacterial effects of garlic cloves, lotus leaves, and ginkgo leaves against a variety of food-borne pathogenic bacterial strains, including *B. cereus* ^55, 56^.

The literature suggests that the active ingredients of plant extracts such as phenolics and flavonoids may bind to the cell surface and penetrate target sites such as the plasma membrane containing membrane-bound enzymes, disrupting the cell wall structure^57^. Thus, among all the tested plant extracts and combinations thereof, the ethanolic extract of ginkgo leaves showed the strongest bactericidal activity, as indicated by the significant reduction in microbial counts and complete inhibition of *B. cereus* growth. Based on these results, selected plant extracts can be considered promising anti-microbials for improving food safety by controlling *B. cereus* counts in food products.

In our previous study, we evaluated the fungal microflora in *Meju* samples fermented with single and/or multiple plant extracts, including lotus leaves, ginkgo leaves, and garlic cloves, at different concentrations (1%, 10%, and a mixture of these extracts in a ratio of 1:1:1). *Meju* samples fermented with individual and/or multiple plant extracts showed the presence of various non-hazardous fungal strains, including *Aspergillus* species, *Mucor* species, *Paecilomyces* species, and *Penicillium chrysogenum*. In contrast, the control *Meju* samples fermented without plant extracts revealed the presence of other fungal microflora such as *Aspergillus ruber* ^58^, which is known to produce aflatoxin B1 and ochratoxin A^59^. The findings of the present study confirm that the fermentation of *Meju* with plant extracts may strongly affect the fungal microflora by eliminating hazardous fungal pathogens through the suppressive effects of the extracts against toxin-producing fungal pathogens, improving the quality of *Meju*.

In addition, we analyzed functional properties of selected plant extracts. The literature and our experimental data show that these plant extracts and their bioactive compounds have various known pharmacological effects^18, 19^. As a reference, there are many other plant components that are directly related to several other functional activities, including anti-microbial activities^18^. Additionally, similar studies have demonstrated the direct and indirect inhibitory effects of several plant extracts and their active ingredients on certain biological activities^60, 61^.

### GC-MS profile of bioactive compounds in *Meju* samples

GC-MS is an analytical method that combines the features of gas-liquid chromatography and mass spectrometry to identify different substances in a variety of samples. GC-MS analysis of plant extract-supplemented *Meju* samples and a control *Meju* sample resulted in the identification of 113 compounds representing 98.44–99.98% of the total extract. All tested *Meju* samples yielded compounds largely composed of alcohols, sugars, amides, furanones, phenolics, terpenoids, steroids, alkaloids, and flavanones, as well as hydrazine and imidazole derivatives and other phytochemicals. It was noted in this study that the percentage of compounds increased in all the tested *Meju* samples as the concentration of plant extracts increased from 1% to 10% (Table S1). A detailed chemical profile of various plant extract-supplemented *Meju* samples and the control *Meju* sample (with no added plant extracts) is presented in Table S1.

Docosenamide (Table S1), an amide compound, was present in all the tested *Meju* samples. After comparing with control *Meju* samples, the 1% plant extract-supplemented samples showed relative areas ranging from 0.12% to 0.14%, whereas 10% plant extract-supplemented *Meju* samples yielded a higher range (0.27–0.89%) of the relative area. Ghazali *et al*.^62^ reported the presence of docosenamide in the root extract of *Ixora coccinea*, which showed anti-microbial effects. Kitaoka *et al*.^63^ isolated and purified laminaribiose from *Euglena gracilis*, a disaccharide important in the field of agriculture as an antiseptic. Laminaribiose (Table S1) was also present in *Meju* samples supplemented with garlic cloves, lotus leaves, or ginkgo leaves as well as in mixed *Meju* samples, but it was absent in the control *Meju* sample. It was found that in the 10% plant extract-supplemented *Meju* samples, the relative peak area was slightly larger (0.19–0.35%) than that for the 1% plant extract-supplemented *Meju* samples (0.07–0.18%). Another key compound that was present in all the tested *Meju* samples is erythritol, a four-carbon sugar alcohol (polyol). Generally recognized as safe, erythritol is used in the food industry as a low-calorie sweetener^64^. The alkaloid *N*-methylasimilobine, which was present only in 1% and 10% lotus leaf extract-supplemented *Meju* samples and in 1% and 10% mixed extract-supplemented *Meju* samples, has been reported to possess acetylcholinesterase inhibitory activity^65^. In our previous study^18^, we reported the presence of *N*-methylasimilobine in the methanol extract of lotus leaves, indicating the potential therapeutic usefulness of this extract.

In addition, control and plant extract-supplemented *Meju* samples contained an aromatic acid component, azaazoniaboratine (Table S1), which has been found to exert an anti-cancer effect^66^. On the other hand, furanone and its various derivatives possessing antioxidant and anti-inflammatory activities^67^ were also present in the *Meju* samples analyzed, indicating the medicinal utility of the *Meju* samples under study. Among furan derivatives, the furan ring is a constituent of several important natural products, including fructofuranose, psicofuranose, sorbofuranose, allofuranose, and many other natural terpenoids.

Other volatile organic acids such as lactic acid, acetic acid, propanoic acid, citric acid, acrylic acid, palmitic acid, and butenedioic acid were also present in all the tested *Meju* samples and can provide an important contribution to the flavor characteristics of various foods while imparting antioxidant and other pharmacologically important activities. These results are in strong agreement with the findings of Tang *et al*.^68^. Propionic acid is a naturally occurring carboxylic acid that inhibits the growth of molds and some bacteria when added to food at concentrations between 0.1% and 1%^69^; the acid was also detected in our *Meju* samples. Moreover, anthraquinone, which has been reported to possess strong anti-microbial properties^70^, was found in abundance in garlic and ginkgo leaf extract-supplemented *Meju* samples and in mixed-extract-supplemented *Meju* samples, suggesting that anthraquinone alone or in combination with other bioactive compounds of *Meju* samples may be responsible for the observed anti-microbial effect. In this study, GC-MS analysis of different *Meju* samples detected the presence of common bioactive compounds and specific compounds because different plant extracts were used for *Meju* production.

## Conclusions

The purpose of this study was to develop a new system for producing novel types of traditional Korean *Meju* products by incorporating various plant extracts with significant biological effects during fermentation. GC-MS analysis of various plant extract-supplemented *Meju* samples identified various bioactive compounds such as organic acids, amino acids, fatty acids, terpenes, phenolics, sterols, alkaloids, flavonoids, and sugars, as well as furans and their derivatives. Moreover, the percentage of various bioactive compounds found in plant extract-supplemented *Meju* samples gradually increased with the concentration of plant extracts during *Meju* fermentation. Fermented soy products such as *Meju* prepared from selected plant extracts derived from garlic cloves, lotus leaves, and ginkgo leaves individually or in combination at different concentrations (1% and 10%) exerted strong inhibitory effects against the growth of *B. cereus*. Moreover, the fermented *Meju* samples incorporated with ginkgo leaf extract (10%) showed considerable tyrosinase and α-glucosidase inhibitory effect compared with traditional *Meju* samples fermented without plant extracts. These findings reinforce the notion that the various biological and functional properties observed for *Meju* samples incorporated with ginkgo leaf extract may be attributed to various biologically active polyphenolic compounds, which may act either individually or synergistically. Based on the abovementioned findings, it can be concluded that these plant extracts can serve as natural additives during the production of functional *Meju* products with acceptable attributes.

## Electronic supplementary material

Supplementary Information
