# Supplementary material for: Development of novel Meju starter culture using plant extracts with reduced Bacillus cereus counts and enhanced functional properties
Source: Sci Rep. 2017 Sep 12;7:11409. doi: 10.1038/s41598-017-09551-0 (PMC5595882; doi:10.1038/s41598-017-09551-0)
Supplement: Supplementary file 1 — Supplementary Information [file 41598_2017_9551_MOESM1_ESM.doc]

**Supplementary Dataset 1**

**Development of novel *Meju* starter culture using plant extracts with reduced *Bacillus cereus* counts and enhanced functional properties**

**Shruti Shukla^1,2^, Juyeon Park^2^, Jung Hyun Park^2^, Jong Suk Lee^3^ & Myunghee Kim^2^,***

^1^Department of Energy and Materials Engineering, Dongguk University-Seoul, 30 Pildong-ro 1-gil, Seoul 04620, Republic of Korea

^2^Department of Food Science and Technology, Yeungnam University, Gyeongsan-si, Gyeongsangbuk-do 38541, Republic of Korea

^3^Department of Food, Nutrition and Cooking, Taegu Science University, Daegu 702-723, Republic of Korea

**Running head:** Functional properties of *Meju* fermented with plant extracts

***To whom correspondence should be addressed:**

**Prof. Myunghee Kim**

Phone: +82 53 8102958; Fax: +82 53 8104662;

E-mail: [foodtech@ynu.ac.kr](mailto:foodtech@ynu.ac.kr)

**Table S1.** Analysis of chemical composition profile of bioactive compounds present in different plant extract added *Meju* samples by GC-MS

| **No.** | **Compounds** | **Retention time** | **Mass spectra (m/z)^2^** | **Relative peak area (%)** | | | | | | | | |
| --- | --- | --- | --- | --- | --- | --- | --- | --- | --- | --- | --- | --- |
|  |  |  |  | **CON** | **GAM1** | **GAM10** | **LOM1** | **LOM10** | **GIM1** | **GIM10** | **MIM1** | **MIM10** |
| **Organic acids** | | | | | | | | | | | | |
| 1 | Lactic acid (PubChem CID: 612) | 9.7 | 45,73, 117, 147, 191.1, 219.1 | 10.15 | 21.98 | 14.31 | 17.15 | 5.48 | 16.8 | 3.4 | 7.24 | 6.01 |
| 2 | Acetic acid | 10.4 | 28, 45, 73, 147, 161, 177, 205 | - | 0.10 | 0.14 | - | - | - | - | - | - |
| 3 | Succinic acid | 23.7 | 45, 73,147, 172, 247.1 | 0.30 | 0.32 | 0.39 | 0.41 | - | 0.26 | - | 0.28 | 0.43 |
| 4 | Propanoic acid (PubChem CID:1032) | 24.7 | 73.1, 103, 117, 133, 147, 189.1, 205.1, 292.1 | - | 0.22 | 0.25 | 0.31 | - | 0.14 | - | 0.11 | 0.51 |
| 5 | 2-Butenedioic acid | 26.0 | 28, 45, 133, 73.1, 147, 245 | 0.23 | 0.16 | - | 0.29 | - | 0.12 | - | 0.10 | 0.35 |
| 6 | Citric acid (PubChem CID:311) | 55.4 | 45, 73, 147, 204 | 0.15 | 0.36 | 0.80 | 0.37 | 0.48 | - | - | - | - |
| 7 | Galactonic acid | 57.7 | 73.1, 103, 147, 217.1, 466 | 0.27 | - | 0.09 | - | - | - | - | - | - |
| 8 | Acrylic acid | 58.4 | 28, 73, 103, 147, 205.1, 305 | - | 0.51 | - | - | - | - | - | 0.10 | - |
| 9 | Gluconic acid | 59.8 | 73, 103, 147.1, 205.1, 242.1, 292.1, 319.1 | - | 0.10 | - | 0.07 | 0.13 | - | - | - | 0.10 |
| 10 | Bis-o-trimethylsilyl-palmitic acid | 81.2 | 43, 73, 103, 147, 175, 203, 239, 313.3, 371, 459.3 | 0.28 |  | - | - | - | - | - | - | 0.22 |
| 11 | 2-Piperidinecarboxylic acid (Pipecolic acid) | 20.5 | 56.1, 84, 103, 186.1 | - | - | - | - | - | - | - | 0.09 | - |
| 12 | Cyclohexene-1-carboxylic acid | 55.5 | 73.1, 147.1, 204.1, 255.1 | - | - | - | - | - | 2.03 | - | 0.20 | 3.99 |
| 13 | 2-Pyrrolidone carboxylic acid | 35.8 | 73, 103, 156.1, 217.1 | - | - | 1.53 | - | - | - | - | - | 1.21 |
| **Fatty acids** | | | | | | | | | | | | |
| 14 | Hexadecanoic acid | 62.2 | 73, 117, 145, 201, 269.2, 313.2 | 3.71 | 0.79 | - | - | - | - | - | - | - |
| 15 | Palmitic acid | 62.25 | 73.1, 117, 145, 201, 313.3 | - | - | - | 1.09 | 8.76 | 1.75 | 8.37 | 10.03 | 2.29 |
| 16 | Propanedioic acid | 17.1 | 45, 73, 147, 233.1 | - | 0.10 | 0.18 | - |  |  |  |  |  |
| 17 | Octadecadienoic acid | 66.4 | 41, 73, 75, 129, 178, 262, 220, 337 | 13.18 | - | - | 4.09 | - | 6.56 | 2.63 | 0.10 | 8.15 |
| 18 | Elaidic acid | 63.5 | 55.1, 74, 97.1, 123.1, 141.1, 222.2, 264.2 | - | - | - | 0.07 | 0.20 | - | 0.34 | 0.21 | 0.24 |
| 19 | Oleic acid (PubChem CID: 445639) | 66.6 | 41, 73, 117, 145, 185.1, 222.2, 264.2, 339 | 6.85 | 0.14 | - | 2.21 | 10.40 | 3.32 | 11.26 | 0.10 | 0.35 |
| 20 | Stearic acid | 67.3 | 43, 73, 117, 145, 201, 297.2, 341.3 | 1.11 | 0.19 | 0.27 | 0.38 | 2.75 |  | 2.18 | 3.61 | 0.73 |
| 21 | Linoleic acid | 63.3 | 67, 95.1, 110, 135.1, 150.1, 263.2, 294 | 0.10 | - | -- | 0.04 | 0.32 | 0.09 | 0.54 | 0.30 | 0.10 |
| 22 | Eicosenoic acid | 73.6 | 73, 129, 331.1, 376.3 | - | - | - | - | - | - | - | 0.18 | 0.23 |
| 23 | Arachidic acid | 75.0 | 43.1, 73.1, 117, 145, 201, 369.3 | - | - | - | - | - | - | - | 0.27 | 0.50 |
| **Sugars** | | | | | | | | | | | | |
| 24 | Fructose | 57.2 | 103, 147, 189, 217.1, 277, 307, 364 | 3.72 | 0.10 | 6.67 | 4.34 |  | 4.59 |  | 2.00 | 5.30 |
| 25 | xylose | 57.6 | 73.1, 103, 147.1, 277.1, 307.2, 364.2 | - | - | - | 3.60 | - | - | - | - | - |
| 26 | Tagatose | 57.6 | 73.3, 103, 147, 189, 217.1, 277.1, 307.2 | 3.18 | 5.69 | 5.51 | - | - | 3.81 | - | 1.78 | - |
| 27 | Sorbose | 55.1 | 28, 73.1, 147.1, 204.1, 437.2 | - | 0.11 | 0.59 | - | - | - | - | - | - |
| 28 | Talose (Mannose) | 57.9 | 73, 103, 147, 205, 319 | 0.50 | - | - | - | - | - | - | 0.12 | - |
| 29 | Galactose | 57.99 | 73.1, 103, 147, 205.1, 319.2 | - | 0.53 | 0.37 | 0.36 | - | - | - | 0.18 | - |
| 30 | Raffinose | 83.69 | 73.1, 103, 147, 191, 217.1, 243.1, 289.1, 361.2 | - | - | - | - | - | 0.17 | - | 0.09 | - |
| 31 | Trehalose | 86.2 | 73, 103, 147, 191.1, 127.1, 361.2 | 1.78 | 0.34 | 0.50 | 1.64 | - | 0.53 | - | 3.56 | 3.91 |
| 32 | Sucrose (PubChem CID: 5988) | 83.0 | 73, 147, 217.1, 271.1, 361.2, 437.2 | 12.46 | 11.94 | 11.88 | 7.74 | - | 16.8 | - | 4.80 | 10.12 |
| 33 | Ribopyranose | 60.5 | 73, 103, 147, 191, 204, 217 | 0.08 | - | - | - | - | 0.10 | - | - | - |
| 34 | Nigerose (PubChem CID: 439512) | 87.3 | 271.1, 480.3, 319.2, 361.2, 480.3 | - | 0.74 | 0.89 | 0.06 | 0.13 | 0.11 | 0.17 | - | 0.21 |
| **Furans and derivatives** | | | | | | | | | | | | |
| 35 | Furanone | 12.2 | 28, 44.1, 56.6, 75, 117,131, 142, 160 | - | 0.09 | - | - | - | - | - | - | - |
| 36 | Fructofuranose | 54.9 | 147, 217.1, 257.0, 437.2 | 0.30 | 0.34 | - | 0.19 | - | 0.22 | - | 0.14 | 0.37 |
| 37 | Psicofuranose (PubChem CID:6427965) | 55.2 | 103, 147, 217.1, 437.2 | 0.78 | 0.43 | 0.53 | 0.47 | - | 0.65 | - | 0.39 | 0.46 |
| 38 | Sorbofuranose | 55.2 | 73, 103, 147.1, 217.1, 437.2 | - | 0.55 | - | 0.23 |  |  |  | - | 0.16 |
| 39 | Allofuranose | 59.9 | 78.1, 103, 147.1, 191.1, 217.1, 246.1, 305.1 | - | 0.15 | - | - | - | 0.16 | - | - | - |
| 40 | Talofuranose | 60.5 | 73, 103, 147, 191, 217, 246.1, 318.1 | - | 0.10 | - | - | - | - | - | - | - |
| 41 | Ribofuranose | 52.1 | 73.1, 147, 189, 271.1, 306.1, 392.2 | - | - | - | - | - | - | - | - | 0.24 |
| 42 | Dihydrofuranno (3,2-H) homochromanon | 92.81 | 28, 73,147.1, 204.1, 259.1 | - | - | - | - | - | 0.04 | - |  | - |
| - **Amino acids** | |  |  |  | - | - | - | - | - | - | - | - |
| 43 | L-Alanine | 11.6 | 28, 45, 59, 73.1, 116.1, 147, 190 | - | 0.07 | 0.12 | - | - | - | - | 0.08 | 0.24 |
| 44 | Glutamic acid | 52.0 | 73, 147, 217.1, 306.1, 392.2 | - | - |  | 0.17 |  |  |  |  |  |
| 45 | Serine | 85.6 | 73.1, 101, 191.1, 218.1, 337.3, 408.3 | 0.21 | - | - | - | - | - | - | - | - |
| 46 | Glutamic acid | 83.4 | 103, 147, 169, 217.1, 289.1, 319.1, 361.2, 437.2 | - | 0.15 | - | - | - | - | - | - | - |
| 47 | L-Threonic acid | 40.0 | 73, 147.1, 220.1, 292.1 | - | - | - | - | - | - | - | - | 0.14 |
| **Alcoholic and terpene compounds** | | | | | | | | | | | | |
| 48 | Silanol | 21.1 | 133.0, 299.1, 314.0 | 2.08 | 1.33 | 1.92 | 1.30 |  | 1.01 | 2.79 | 0.93 | 0.79 |
| 49 | Glycerol | 21.5 | 103, 117,133,147, 205, 218.1 | 8.17 | 3.00 | 3.40 | 1.85 | 1.95 | 5.21 |  | 5.70 | 6.44 |
| 50 | Erythritol | 35.8 | 45.1, 73, 103,129,147, 189,217,307 | 1.20 | 1.90 | 2.1 | 0.15 | 3.2 | 1.50 | 2.5 | 1.05 | 5.99 |
| 51 | Xylitol (PubChem CID: 6912) | 51.5 | 103, 147, 189.1, 217.1, 243.1, 277.1 | 1.96 | 0.86 | 0.89 | 5.12 |  | 0.85 |  | 2.04 | 3.40 |
| 52 | Glucitol | 58.9 | 73.1, 103, 147, 205.1, 319 | 10.09 | 11.59 | 18.06 | 10.47 |  | 10.14 |  | 4.49 | 8.72 |
| 53 | Inositol | 59.6 | 73.1, 103, 147, 205, 319.2 | 0.24 | 0.33 | 0.33 | 0.30 |  | 0.41 |  | 0.17 |  |
| 54 | Nootkatone | 21.4 | 73.1, 103, 117, 147, 205, 218.1 | - | - | - | - | - | 0.16 | 0.60 | - | - |
| 55 | Kokoononol/Friedooleanan/ Tetracosanoic acid | 89.1 | 73.1, 117, 145, 304.1, 425.4 | - | - | - | - | - | - | - | 0.10 | 0.59 |
| **Sterols** | | | | | | | | | | | | |
| 56 | Stigmasterol | 102.2 | 55.1, 83.1, 129, 159, 255, 355.3, 394.4, 484.5 | 0.13 | 0.13 | 0.13 | 0.12 | 0.39 | 0.13 | 0.35 | - | - |
| 57 | Sitosterol trimethylsilyl ether | 105.1 | 73.1, 129.1, 357.4, 396.4, 486.4 | 0.27 | 0.26 | 0.28 | 0.30 | 1.55 |  | 0.94 | 0.27 | 0.29 |
| 58 | Campesterol/ Ergot-5-en-3-ol | 99.9 | 43.1, 81, 107, 159.1, 255.1, 289.1, 404.4 | - | - | - | 0.02 | 0.54 | 0.03 | 0.22 | - | - |
| 59 | Androstan | 102.2 | 73.1, 147, 217.1, 318.1, 387.2 | - | - | 0.27 | 0.20 | - | - | - | - | 0.38 |
| **Phenolics** | | | | | | | | | | | | |
| 60 | Tocopherol (PubChem CID: 14985) | 90.4 | 73, 209.1, 249.1, 474.44 | 0.16 | 0.14 | 0.16 | 0.33 | 0.56 | 0.17 | 0.26 | 0.17 | 0.21 |
| 61 | Gentibiose | 98.2 | 73, 103, 129, 204.1, 243.1, 361.1 | 0.11 | 0.12 | 0.13 | 0.22 | 0.29 | 0.15 | 0.25 | - | 0.24 |
| 62 | 1-[2,4,6-tris (trimethylsiloxy)phenyl]-3-[methoxy-4-(trimethylsiloxy)phenyl | 99.2 | 73, 204.1, 575.2 | - | - | 0.09 | 0.26 | - | - | - | - | 0.40 |
| **Alkaloids** | | | | | | | | | | | | |
| 64 | Imidazole (PubChem CID: 795) | 37.2 | 56.1, 84, 157.9, 174.1, 276.1 | - | 0.15 | - | 0.15 | - | - | - | - | - |
| 65 | Morphinanedienone | 103.8 | 73, 129. 159.1, 227.1, 317.2, 369.4, 527.5 |  | - | - | - | - | 0.14 | 0.48 | - | 0.21 |
| 66 | 2-[(Trimethylsilylmethylamino) (methylthio)] methylene-1,3-indandione | 58.40 | 73, 305.1 | 0.20 | - | - | - | - | 0.20 | - | - | 0.30 |
| 67 | N- Methylasimilobine (PubChem CID:197017) | 73.9 | 42.1, 280.1, 178.1, 223.1, 250.1 | - | - | - | 0.08 | 0.35 | - | - | 0.02 | 0..32 |
| 68 | 6,7-Dimethoxy tetrahydropyrrolo quinolin | 104.5 | 28, 73.1, 157.1, 306.2 | - | - | 0.60 |  | 0.80 | - | - | - | - |
| 69 | Dihydrocorynantheine | 90.4 | 73, 267.1, 368.2, 474.2, 650.4 | - | - | - | - | - | - | - | - | 0.32 |
| 70 | Monooleylglycerol trimethylsilyl ether (Benzoyldehydronuciferine) | 86.8 | 73, 103, 129, 203, 397 | 0.41 | - | - | 0.13 | 1.21 | - | - | 1.78 | 1.88 |
| **Flavonoids** | | | | | | | | | | | | |
| 71 | 4-dihydro-1H-quinolin | 75.5 | 73, 109.1, 165, 191.1, 294.1, 352.2 | - | - | - | - | 1.24 | - | - | - | 0.18 |
| 72 | Trimethylsilyl 6-heptadecylginkgolate | 76.6 | 28, 73, 103.1, 219.1, 361.2, 503.4 | - | - | - | - |  | 0.09 | - | - | 0.25 |
| 73 | Dihydroxy-5-Methoxy-3-Methylan-thraquinone) | 95.7 | 73, 191.3, 355.1, 398.1, 428.1 | - | 0.06 | 0.06 | 0.12 |  | 0.06 | - | - | - |
| 74 | Quinic acid (PubChem CID: 6508) | 56.80 | 73.1, 103, 147, 191.1, 255.1, 319, 345.1 | - | - | - | - | - | 0.17 | 1.12 | 0.10 | 1.88 |
| 75 | Antharaquinone | 92.9 | 223.1, 263.1, 488.4 | 0.27 | 0.23 | 0.28 | - | - | 0.29 | 0.32 | - | 0.21 |
| **Other important compounds** | | | | | | | | | | | | |
| 76 | 2,3-butandiol | 8.4 | 28, 73, 117, 147 | 0.13 | 0.09 | 0.11 | 0.11 |  | 0.07 | - | 0.15 | 0.44 |
| 77 | Azaazoniaboratine | 55.7 | 103, 147, 191, 217.1, 260.1, 318.1, 343.1, 374.2, 433.2 | 2.98 | 4.93 | - | 5.86 | - | 4.90 | - | 2.30 | 3.76 |
| 78 | 8-Allyl-2 amino 6- methyl imidazotriazine | 16.5 | 73, 101, 117, 131, 147, 205.1 | - | - | - | - | 1.57 | - | - | - | - |
| 79 | Pinitol | 55.7 | 147, 191.1, 217.1, 260.1, 318.1, 433.2 | - | - | 3.77 | 4.56 |  | - | - | - | 3.80 |
| 80 | Glucosamine | 58.5 | 147.1, 203.1, 304.1 | 0.15 | 0.12 | 0.41 | 0.11 | - | 0.16 | - | - | - |
| 81 | Cobalt | 57.9 |  | - | - | - | - | - | 0.47 | - | - | 0.54 |
| 82 | Inositol | 59.6 | 73.1, 103, 147, 205, 319.2 | 0.24 | 0.33 | 0.33 | 0.30 | - | 0.41 | - | 0.17 | - |
| 83 | 3-Bromo-5-ethoxy-4-hydroxybenzaldehyde | 61.05 | 28.1, 73.1, 103.1, 147.1, 191.1, 217.1, 246.1, 318.2 | - | - | - | - | - | 0.07 | - | - | - |
| 84 | Myo-Inositol | 63.1 | 45, 73.1, 103, 147, 191.1, 217.1, 265.1, 305.1, 376.2 | 0.25 | 0.31 | 0.82 | 0.63 | - | 0.20 | - | 0.12 | 0.80 |
| 85 | Phenol/ Tyramine/tocosine | 76.3 | 108, 133, 302.3 | - | - | - | - | - | 0.03 | 0.62 | - | - |
| 86 | Phenol/(2-Amino-5-methylpyridine) | 76.8 | 108, 147, 302.3 | - | - | - | - | - | 0.05 | 1.00 | - | - |
| 87 | Phenol/M-N-Undecylphenol | 84.7 | 108, 147.1, 330.3 | - | - | - | - | - | 0.19 | 3.77 | - | - |
| 88 | Neoechinulin | 94.1 | 44, 73, 99, 182.1, 254.1, 361.1 | - | - | - | - | - | 0.12 | 0.27 | - | - |
| 89 | Tryptophan (PubChem CID: 6305) | 65.7 | 73, 202.1 | 0.12 | - | 0.11 | 0.09 | - | - | - | - | - |
| 90 | 2- methyl-4-oxosparteine | 69.2 | 98, 154, 164, 191, 194, 219, 262, 305.1 | 0.15 | - | - | - | - | - | - | - | - |
| 91 | Piperidine | 69.2 | 98, 154, 164, 191, 194, 219, 262, 305.1 | 0.15 | - | - | - | - | 0.09 | - | - | - |
| 92 | Dimethoxy piperidylpodocarpatriene (Picolinyl) | 81.2 | 43,73, 103.1, 147, 203.1, 371.3,  459.4 | - | 0.23 | - | - | - | - | - | - | - |
| 93 | Armidow/Armoslip | 71.1 | 41.1, 59, 126.1, 281.3 | - | - | - | - | - | - | - | 0.15 | - |
| 94 | Cellobiose | 86.0 | 73,103, 147.1, 204.1, 319.2, 361.2 | 0.13 | - | - | - | - | - | - | - | - |
| 95 | D-Glucopyranoside | 86.6 | 73, 147.1, 204.1 | 2.24 | 3.53 | - | - | - | 2.61 | - | - | - |
| 96 | Benzopyranol | 91.4 | 136.9, 177.1, 402.4 | - | - | - | - | 0.63 |  | 0.63 | - | - |
| 97 | Simulansamide | 86.8 | 73.1, 103, 129, 204.1, 361.2, 397.3, 485.4 | - | - | - | - | - | 0.34 | - | - | 0.36 |
| 98 | Docosenamide/Erucylamide (PubChem CID: 5365371) | 87.0 | 29, 59, 83, 126, 154, 337.3 | 0.14 | 0.12 | 0.27 | 0.15 | 0.75 | 0.12 | 0.66 | 0.33 | 0.89 |
| 100 | Palatinose | 87.2 | 103.1, 147, 204.1, 271.1, 319.1, 361.0, 480.3 | 0.26 | 0.03 | 0.80 | 0.51 | - | 0.16 | - | - | 1.17 |
| 101 | Fucopyranose | 89.0 | 73, 147, 204.1 | 1.18 | 2.63 | - | - | - | - | - | - | - |
| 102 | Maltotriose | 89.4 | 103.1, 147, 204.1, 243.1, 361.1, 433.2, 479.2 | - | 0.25 | - | - | - | - | - | - | - |
| 103 | Ascorbic acid | 99.2 | 73, 129.1, 204.1, 597.4, 361.1 | - | - | - | - | - | 0.09 | 0.12 | 0.04 | 0.14 |
| 104 | Maltose | 89.9 | 73, 103.1, 147, 204.1, 243, 271.1, 319, 361.2 | 0.14 | - | - | 0.13 | - | 0.13 | - | - | - |
| 105 | Laminaribiose (PubChem CID:439637) | 90.0 | 28, 73, 103, 147.1, 204.1, 361.2 | - | - | 0.28 | 0.07 | 0.35 | 0.18 | 0.26 | 0.09 | 0.17 |
| 106 | 6,7-di (trimethylsilyloxy) flavone | 90.6 | 28, 73, 184.1, 204.1, 208, 398.1 | 0.29 | 0.32 | 0.41 | 0.57 | - | 0.23 | - | 0.24 | 0.30 |
| 107 | Glucitol | 90.8 | 73.1, 103, 147.1, 204.1, 319.1, 361.2 | 0.13 | - | - | - | - | - | - | 0.09 | 0.17 |
| 108 | Spiro cyclopropane (PubChem CID:14809277) | 91.3 | 147, 129, 204.1, 361.2 | 0.22 | 0.39 | 2.67 | 0.56 | - | 0.28 | - | 0.71 | 1.21 |
| 109 | Methanofutoazone | 91.8 | 73.1, 192, 414.1 | 0.22 | 0.27 | - | 0.43 | - |  | - | - | - |
| 110 | Methoxy-1-naphtholestratriene | 91.86 | 28.1, 73, 192, 414.1 | - | - | 0.21 | - | - | 0.18 | - | - | - |
| 111 | Ethyl 10, 11-o-Isopropylidene-2-[1-hydroxy-2-(trtrahydropyranyloxy)propyl]undecan | 91.9 |  | - | - | - | - | - | 0.03 | - | - | 0.11 |
| 112 | Galactinol/(nonakis trimethylsilyl ether)/ Nonacosan | 92.8 | 73.1, 103, 147.1, 204.1 | - | - | - | 0.11 | 0.42 | - | - | - | - |
| 113 | Biperiden (PubChem CID: 2381) | 69.4 | 43, 73, 117, 145, 201, 341.0 | - | - | - | - | - | 0.04 | 0.12 | 0.07 | - |
| 114 | Ergostoxytrimethyl silane | 101.9 | 95, 129, 343.3, 382.3, 472.4 | 0.18 | 0.15 | 0.17 | 0.17 | - | 0.19 | - | 0.15 | - |

CON: *Meju* without extracts (traditional *Meju*); GAM1: *Meju* with garlic extract at 1% concentration; GAM10: *Meju* with garlic extract at 10% concentration; LOM1: *Meju* with lotus extract at 1% concentration; LOM10: *Meju* with lotus extract at 10% concentration; GIM1: *Meju* with *ginkgo* extract at 1% concentration; GIM10: *Meju* with *ginkgo* extract at 10% concentration; MIM1: *Meju* with mixture extract (1:1:1) at 1% concentration; MIM10: *Meju* with mixture extract (1:1:1) at 10% concentration. As instructed, yellow highlighted 17 important chemical compounds presented in this table (PubChem CID) have also been matched with [NCBI PubChem Compound Database](http://www.ncbi.nlm.nih.gov/pccompound) following the guideline linked at <http://www.elsevier.com/PubChem>.


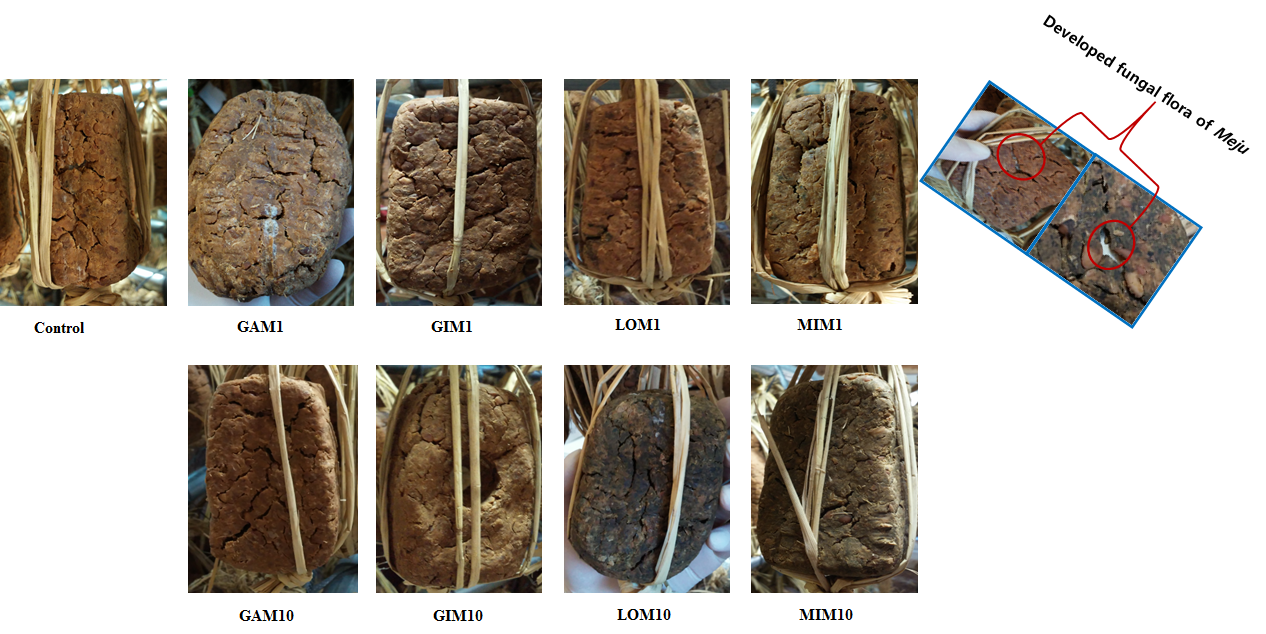


**Fig. S1.** *Meju* samples produced by the addition of plant extracts.

**Section 1**

**Material and Methods**

**Inhibitory spectrum of plant extracts against *B. cereus***

***Determination of diameter of inhibitory zones.*** In this assay, *B. cereus* inhibitory activities of selected plant extracts were analyzed using the disc and agar well diffusion methods. *B. cereus* was grown in nutrient broth and incubated at 37˚C for 18-24 h. After proper bacterial growth, bacterial culture was diluted with peptone water to adjust proper colony forming units 10^7^ cfu/mL after that a suspension of diluted bacterial culture (100 µl) was spread onto plate count agar, followed by drying under a clean bench for a few minutes. Then, 50 µl of each plant extract solution (1% and 10%) of *A. sativum*, *G. biloba*, and *N. nucifera* was introduced to the disc and wells on the agar plates, followed by 18-24 h of incubation at 37˚C. The plates were examined for their zones of inhibition against *B. cereus* measured in millimeters.

***Determination of minimum inhibitory concentration (MIC) against B. cereus.*** Minimum inhibitory concentration microplate assay was used to determine the lowest concentrations of all tested extract samples (*A. sativum*, *G. biloba*, and *N. nucifera*) and their combined ratio to inhibit growth of *B. cereus* according to the procedure developed by Shiu and Gibbons, 2006. Briefly, 100 µl of sterile distilled water was aliquoted into the 96-well sterile plate. Ethanolic extracts of each plant and their combined extract ratio (10 mg/mL) were pipetted into the wells of the microplate. Serial dilutions were performed in the downward direction, resulting in reduced tested sample concentrations. Following serial dilutions, 100 µl of standard culture (10^7^ CFU/mL) was dispensed into each well of a 96-well sterile plate. The plate was sealed and incubated at 37˚C for 24 h for *B. cereus*. Negative controls were prepared using the same solvent employed to dissolve the samples. Following incubation of all tested extracts with *B. cereus*, MIC was determined. To visualize bacterial growth, 40 µl of *p*-iodonitrotetrazolium violet (INT) (0.04 mg/mL) was added to each well, and the plate was incubated at RT for 6 h. Following incubation, the plate was examined for any color change. The obtained results were recorded and analyzed using the Excel program.

**Results and Discussions**

**Inhibitory spectra of plant extracts against *B. cereus.*** *In vitro* inhibitory activities of ethanolic extracts of *A. sativum*, *G. biloba*, and *N. nucifera* and their mixed combination (1:1:1 ratio) against *B. cereus* KCCM 40935 were qualitatively and quantitatively assessed based on the presence of inhibition zones and MIC values. According to Tables 3, ethanolic crude extracts of *N. nucifera* at a concentration of 10% exhibited anti-bacterial effects against *B. cereus* KCCM 40935 with an inhibition zone diameter of 11 mm (Table S2). Ethanol extract of *G. biloba* exerted anti-bacterial activity against the tested bacterium. The inhibition zone diameters of ethanolic extract of *G. biloba* at different concentrations (1% and 10%) against *B. cereus* were 11 and 13 mm, respectively (Table S2). Ethanol extract of *A. sativum* exhibited anti-bacterial activity against *B. cereus* with inhibition zone diameters ranging from 7-9 mm (Table S2). Tetracycline was used as standard drug (10 µg/ mL) and showed an inhibition zone diameter of 21 mm. The inhibition zones and MIC values of the tested extracts and their combinations used for *Meju* production against *B. cereus* ranged from 7–12 mm and 350–5000 μg/mL, respectively. The 5% DMSO control used in this study did not inhibit growth of the test bacterium. The MIC values of *Meju* samples added with plant extracts (*G. biloba*, *N. nucifera*, and mixture of all extracts) against *B. cereus* KCCM 40935 were in the range of 1500-3250 mg/mL (Table S2), whereas the *A. sativum* sample showed ineffective MIC results (12500 mg/mL) (Table S2). These MIC results were in accordance with the inhibitory zone results, confirming that only extracts derived from *G. biloba*, *N. nucifera*, and their mixed combinations had inhibitory effects against *B. cereus* KCCM 40935.

**Table S2. Inhibitory activities (zone of inhibition and MIC) of selected plant extracts against *B. cereus*.**

| **Sample** | **Concentration tested** | **Inhibitory zone (mm)** | **MIC (µg/mL)** |
| --- | --- | --- | --- |
| *N. nucifera* (Leaf) | 1 % | 10± 0.01 | 3250 |
|  | 10 % | 13± 0.03 |  |
| *Ginkgo biloba* (Leaf) | 1 % | 10 ± 0.085 | 1500 |
|  | 10 % | 17± 0.06 |  |
| Garlic (Clove) | 1 % | 1 ± 0.04 | 12500 |
|  | 10 % | 7± 0.86 |  |
| Mixture (1:1:1) | 1 % | 0 ± 0.02 | 3250 |
|  | 10 % | 14 ± 0.27 |  |
| Tetracycline | (10 µg/mL) | 21 ± 0.16 | 25 |


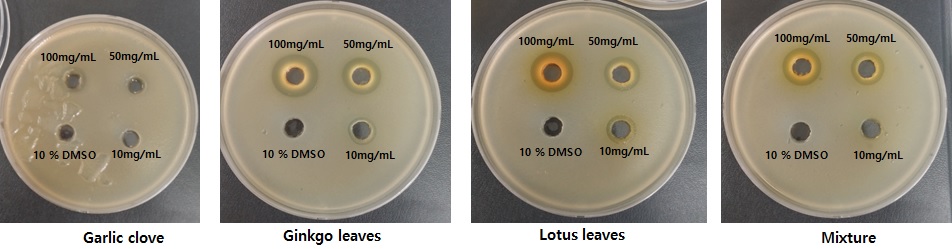
**Fig. S2.** Inhibitory activities in terms of zone of inhibition of selected plant extracts against *B. cereus*.

**Reference**

Shiu, W. K. P. & Gibbons, S. Anti-staphylococcal acylphloroglucinols from *Hypericum beanii*. *Phytochemistry* **67**, 2568-2572 (2006).

**
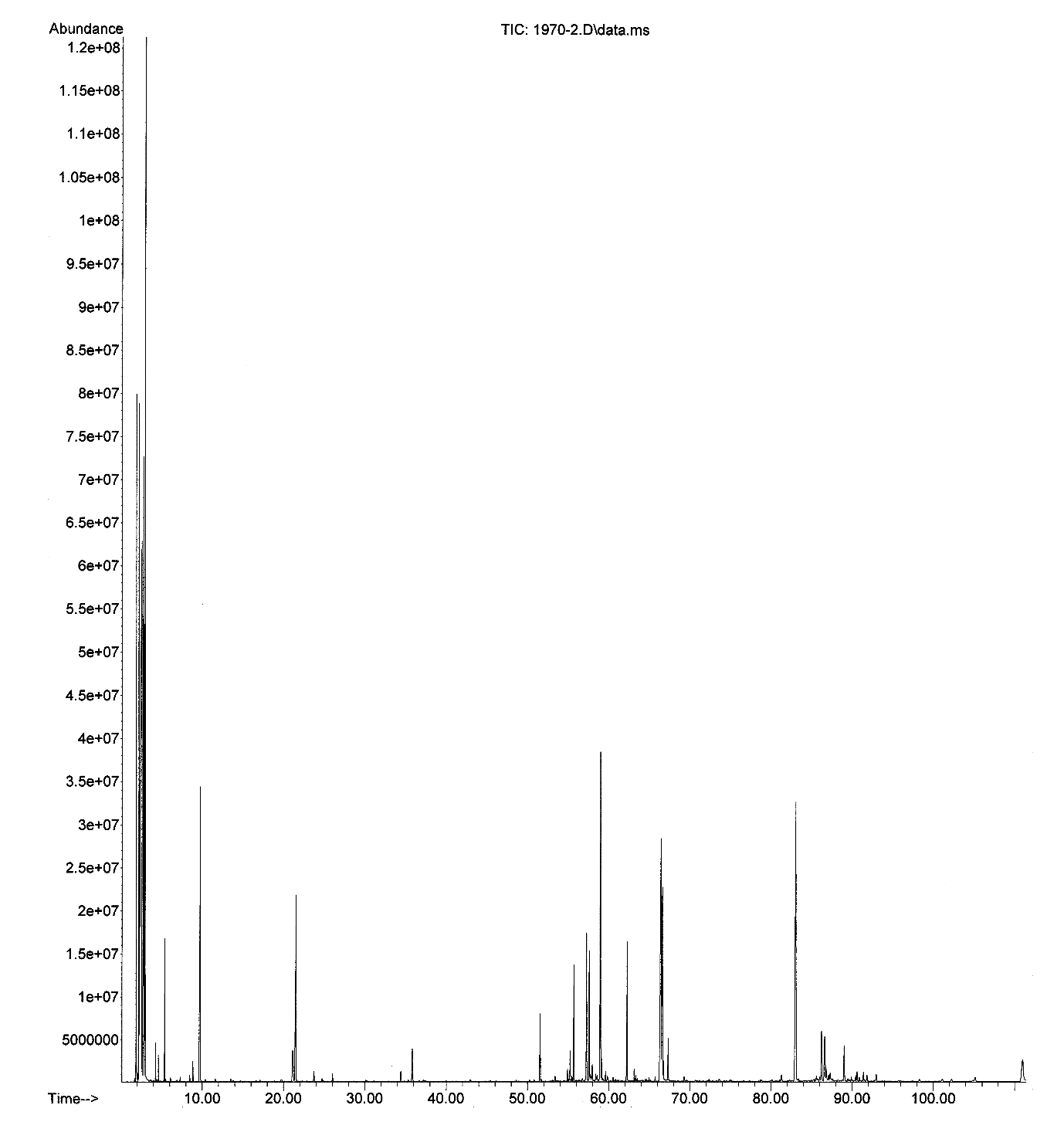
**

**Fig. S3. The GC-MS chromatogram of sample M2 (Control Meju).**


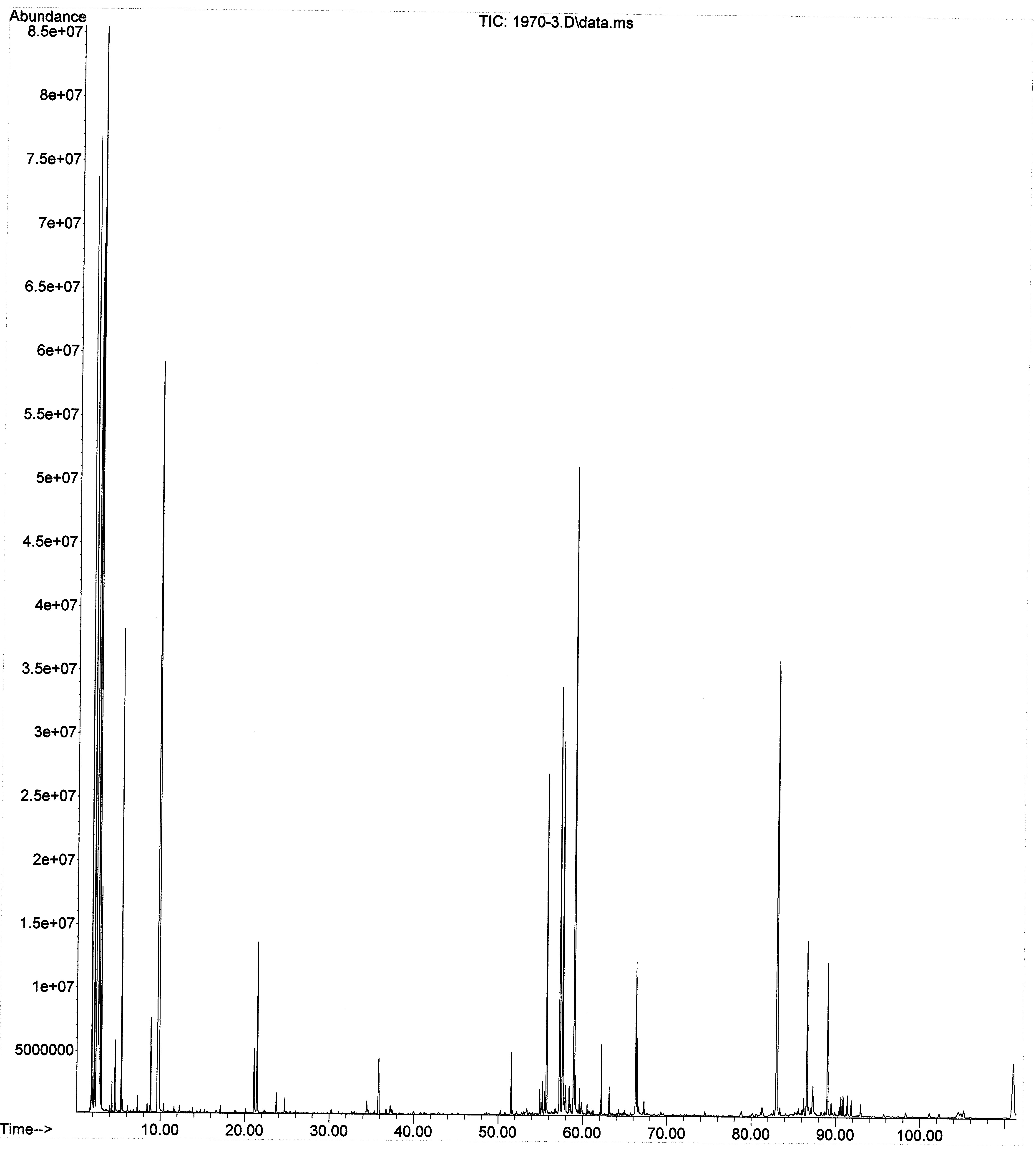


**Fig. S4. The GC-MS chromatogram of sample M4** (**GAM1 Meju).**


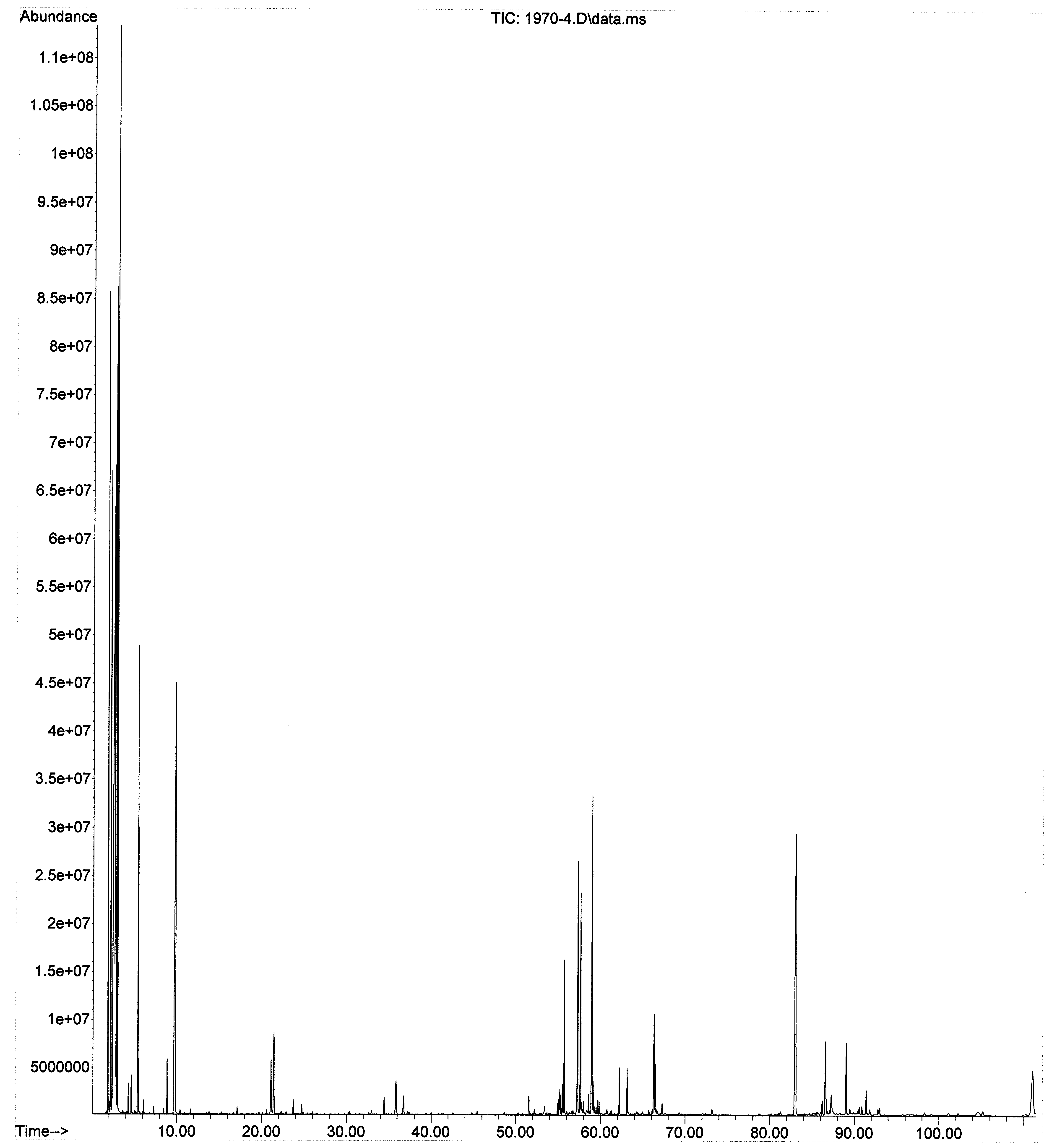


**Fig. S5. The GC-MS chromatogram of sample M6 (GAM10 Meju).**


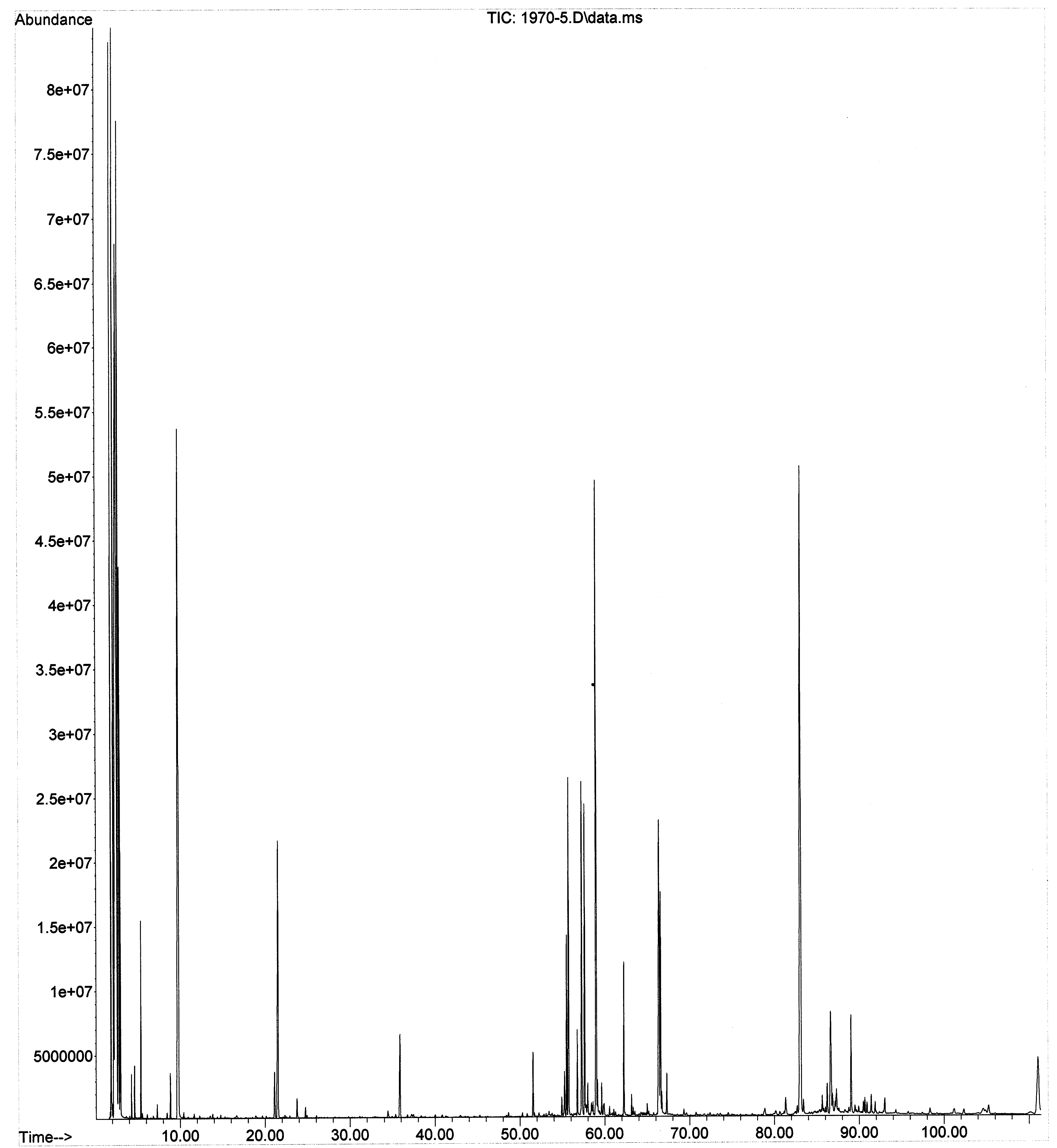


**Fig. S6. The GC-MS chromatogram of sample M8 (LOM1 Meju).**


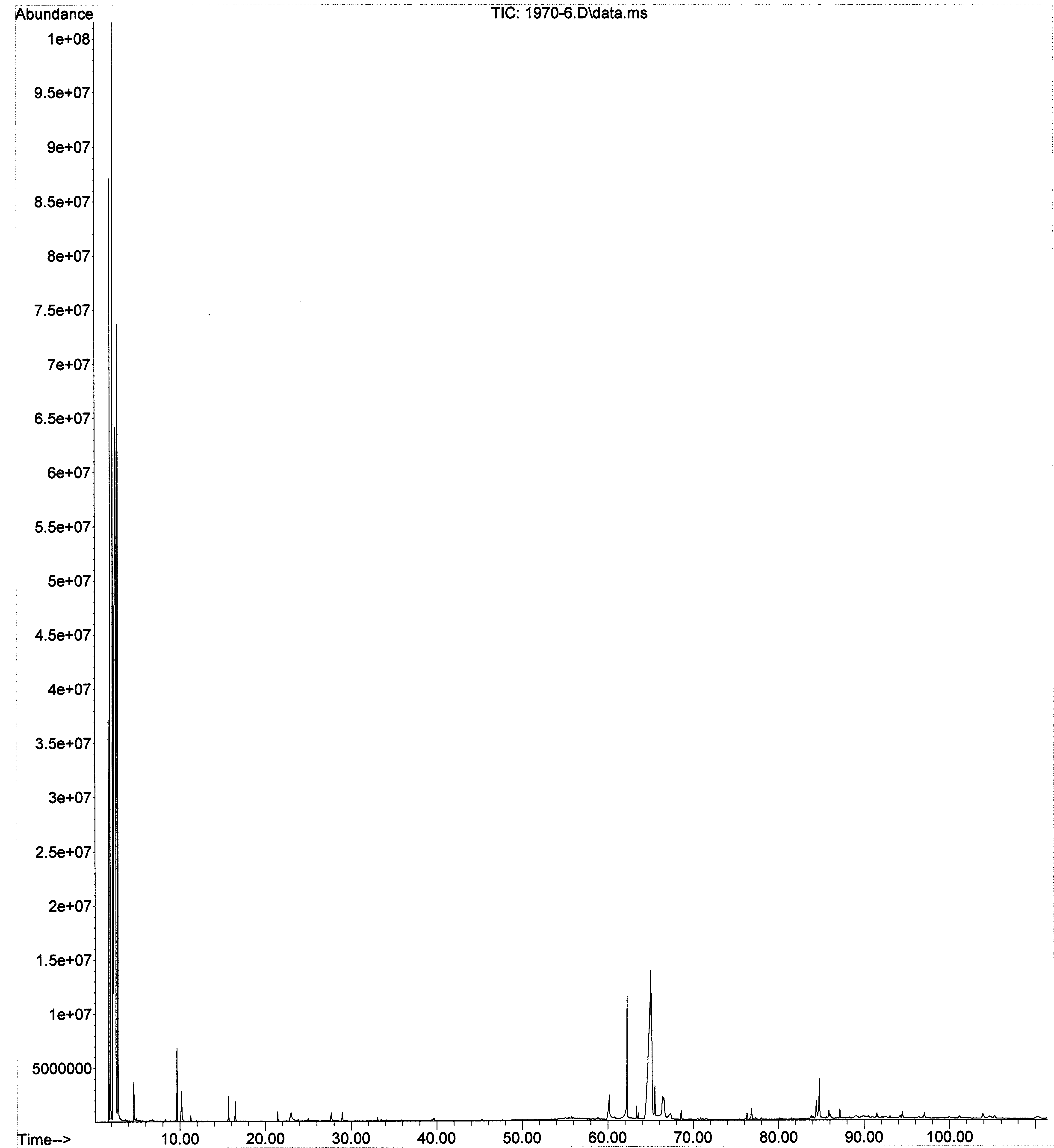


**Fig. S7. The GC-MS chromatogram of sample M10 (LOM1 Meju).**


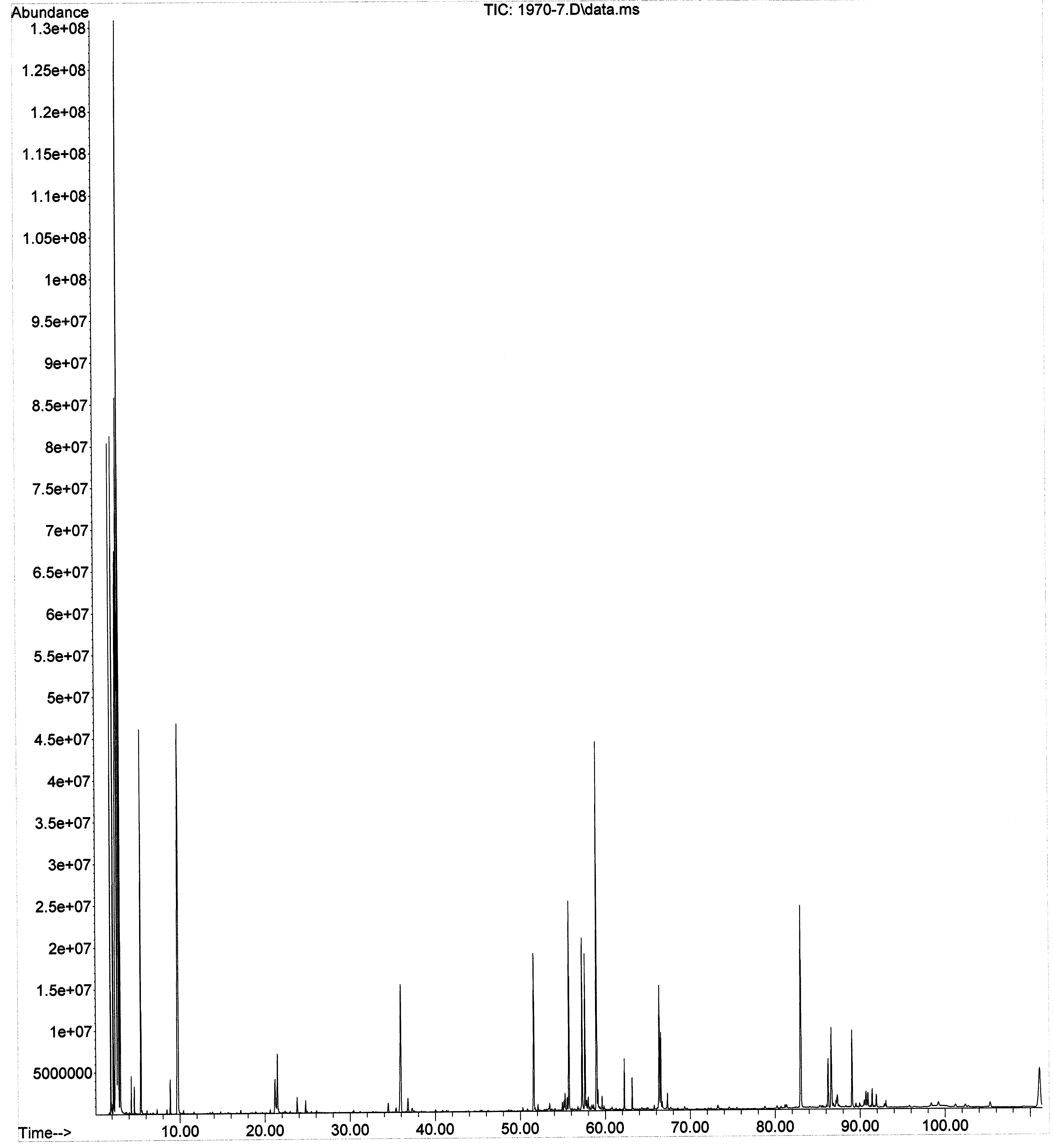


**Fig. S8. The GC-MS chromatogram of sample M12 (GIM1 Meju).**


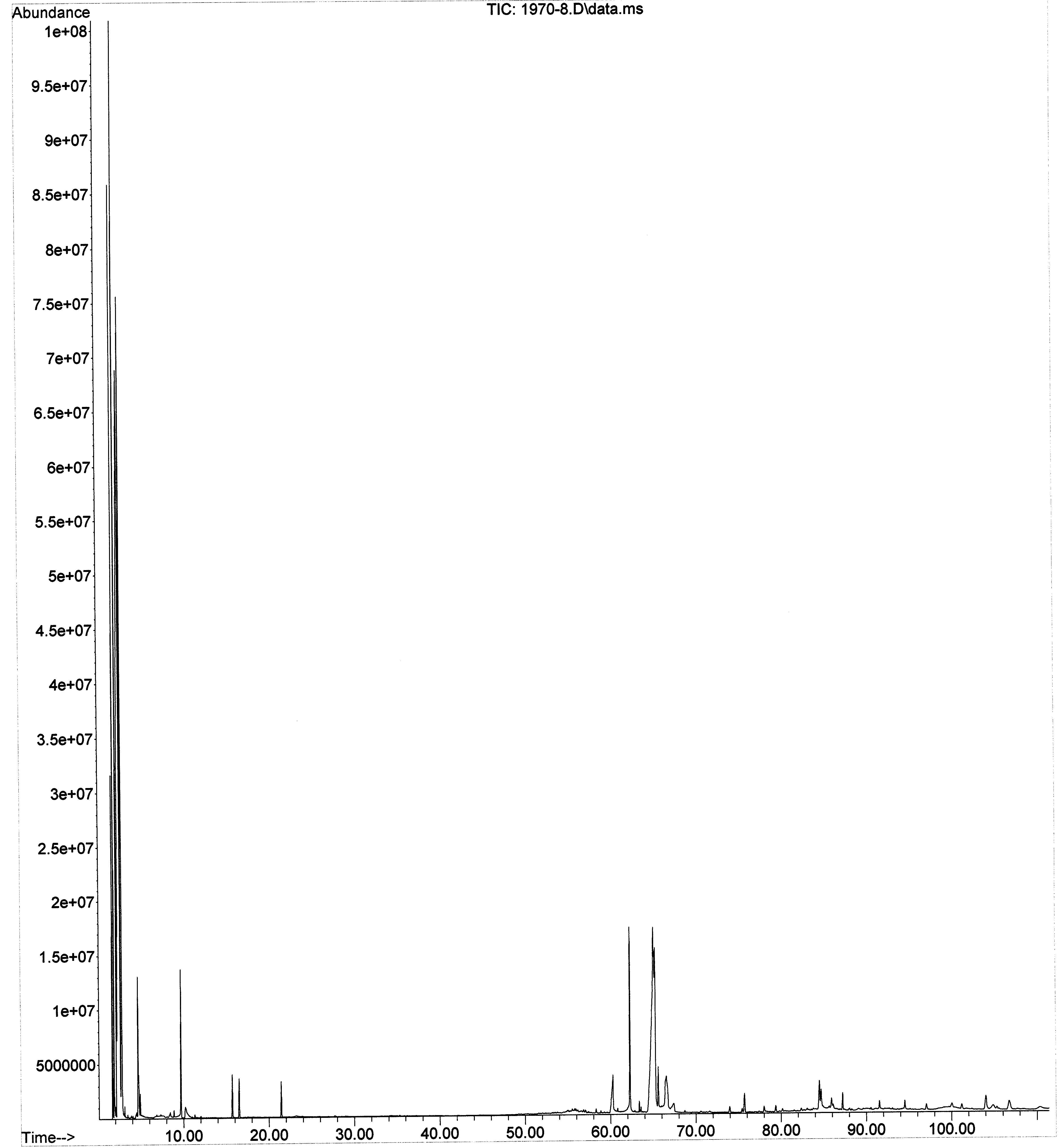


**Fig. S9. The GC-MS chromatogram of sample M14 (GIM10 Meju).**


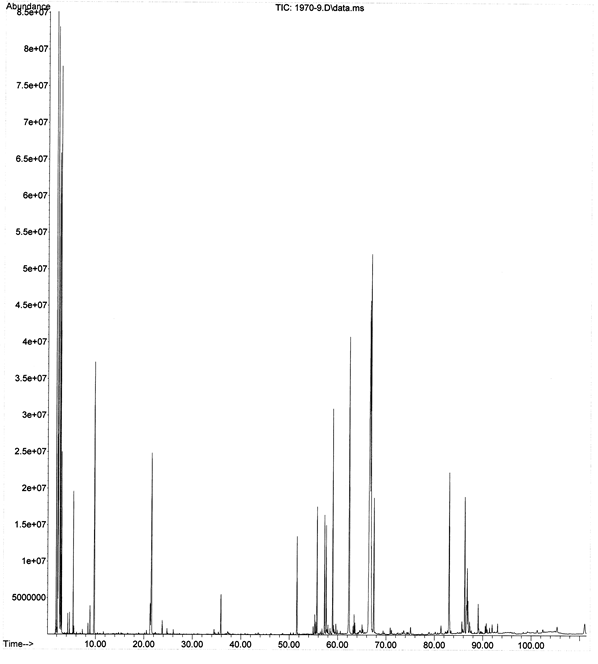


**Fig. S10. The GC-MS chromatogram of sample M16 (MIM1 Meju).**


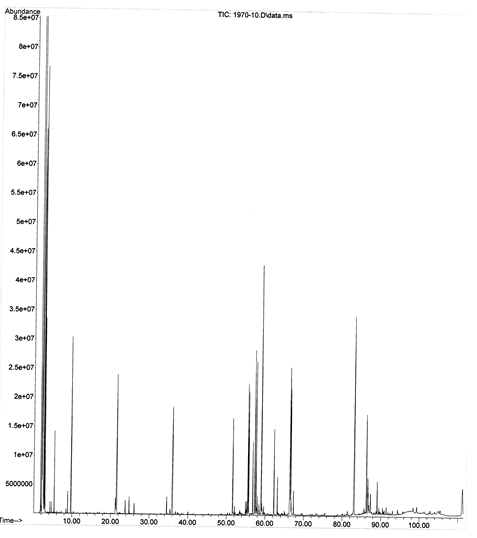


**Fig. S11. The GC-MS chromatogram of sample M18** (**MIM10 Meju).**
